# Intra-Relations of Biochemical Profiles Defining the Germination Potential of Medium-Term Cold-Stored Maize (*Zea mays* L.) Seeds

**DOI:** 10.3390/plants15162437

**Published:** 2026-08-11

**Authors:** Natalija Kravic, Sladjana Zilic, Jelena Vukadinovic, Tanja Petrovic, Marija Milivojevic, Anika Kovincic, Snezana Mladenovic Drinic, Jelena Srdic, Marijana Simic, Vojka Babic, Violeta Andjelkovic

**Affiliations:** Maize Research Institute Zemun Polje, Slobodana Bajica 1, 11185 Belgrade, Serbia; szilic@mrizp.rs (S.Z.); mesarovicj16@gmail.com (J.V.); ptanja@mrizp.rs (T.P.); mmarija@mrizp.rs (M.M.); anisavic@mrizp.rs (A.K.); drinicsnezana2@gmail.com (S.M.D.); jsrdic@mrizp.rs (J.S.); marijana.simic@mrizp.rs (M.S.); vbabic@mrizp.rs (V.B.); violeta@mrizp.rs (V.A.)

**Keywords:** biochemical coordination, ex situ conservation, intracellular glassy matrix, metabolic network, natural seed ageing, physiological vigour, redox homeostasis, *Zea mays* L.

## Abstract

Understanding the biochemical networks governing seed longevity is essential for germplasm conservation. Integrating germination potential with biochemical profiling elucidates metabolic transitions during decadal cold storage. As phenotypic evidence of ageing, extended cold storage induces a uniform decline in germination energy, reaching a terminal “floor effect” characterised by the complete exhaustion of repair-and-defence strategies, whilst residual germination capacity averages 21%. Metabolic profiling reveals that prolonged storage causes systemic decoupling of the protein–sugar axis, leading to the loss of protein-dependent vigour. The structural collapse of the proteomic architecture—manifested by the decoupling of embryo albumin-led coordination and endosperm α-zein dynamics—induces a transition to a low-vigour state, paralleled by carbohydrate shifts consistent with glassy matrix destabilisation, implicitly linking the displacement of sucrose-dominated stability to the molecular crowding of reducing sugars. Re-anchoring germination potential from primary metabolic drivers to soluble-free phenolics prompts a compensatory antioxidant reorganisation; under conditions of phenolic matrix degradation and carotenoid exhaustion, the observed stability of total antioxidant capacity likely reflects scavenging sustained by phenolic degradation products rather than native, intact protective mechanisms. Ultimately, seed ageing manifests as a genotype-specific systemic reconfiguration of biochemical coordination, where the physiological state of the cytoplasmic matrix and the maintenance of redox homeostasis determine residual viability, thereby defining the critical benchmarks for germplasm longevity.

## 1. Introduction

The preservation of genetic diversity within plant genetic resources, particularly crop landraces, is fundamental for mitigating genetic erosion and developing climate-resilient, high-yield cultivars. The challenge of ex situ conservation lies in maintaining long-term seed vitality—specifically germination energy and capacity—while minimizing the progressive loss of genetic integrity over decades [1,2]. Orthodox seeds acquire desiccation tolerance during maturation, the late phase of embryogenesis, by activating a series of protective processes and highly efficient biological mechanisms [3], while simultaneously acquiring longevity, defined as the duration over which they remain viable. This ability to remain in a quiescent state at seed moisture contents below 8% (fresh weight basis) for extended periods significantly enhances their storage stability under medium- and long-term gene bank conditions [4]. However, seed longevity is not a static trait; instead, it represents a dynamic characteristic governed by internal features—chemical composition, metabolic signalling, and germination phenology—and modulated by complex molecular protective and reparative processes [5,6].

Even under optimal storage conditions, seeds undergo irreversible “dry ageing.” This cumulative process causes cellular damage that leads to delayed seedling emergence, increased susceptibility to stress, and ultimately, complete loss of viability [7]. Recent evidence suggests that the mechanisms of decay in the non-glassy, relatively moist state may differ significantly from those in the dry state, where restricted cellular metabolic activity and specific oxidative stress patterns determine survival [8]. Hence, extrapolating seed lifespan from short-term “wet ageing” (accelerated deterioration) to the physiological reality of decadal storage is biologically questionable [9].

Emerging trends indicate that biochemical composition, in terms of total protein, carbohydrates, and antioxidants, provides valuable insights into the susceptibility of seeds to ageing, further emphasizing that the organisation of cellular structures is a primary determinant of longevity in desiccated germplasm [7]. Consequently, divergent structural conformations within the cellular matrix promote distinctive ageing mechanisms in dry cells [10], particularly under the standardised preservation conditions used by most seed banks [11], which may potentially accelerate the rate of viability decline. Furthermore, a holistic comparison of fresh versus long-term stored accessions reveals differences in physiological performance that standard monitoring—while effective at registering the final collapse of vigour—cannot explain [12,13]. By contrasting the metabolic profiles of fresh seeds with those subjected to decadal storage, it becomes possible to identify the specific biochemical markers of vigour that erode long before germination capacity fails. While these metabolic pathways are individually recognised [14], a fundamental knowledge gap persists in the systemic integration of these processes; it remains unclear how the interplay of the proteomic, carbohydrate, and antioxidant networks orchestrates the transition from physiological quiescence to the diminished germination potential observed in such aged accessions.

Despite the importance of seed longevity for efficient ex situ germplasm conservation and crop improvement [15], few studies have systematically compared seed lifespan across different accessions within a species [16,17], and even fewer have examined such long-term stored material [5,16,18]. The Maize Research Institute Zemun Polje (MRIZP) gene bank, housing one of the world’s largest collections—an impressive repository of 5806 accessions—offers a unique longitudinal window into these ageing dynamics.

Building upon our previous observations of maize landrace longevity [19], the present study investigates the systemic reorganization of metabolic networks during 37 years of natural ageing. Our objectives are: (1) to assess the germination energy and capacity, thereby defining the absolute germination potential of aged versus fresh seed samples; (2) to elucidate the functional internal dynamics and intra-network coordination of biochemical markers, using these relationships to map the systemic landscape of viability loss; and (3) to evaluate the potential of these biochemical profiles as physiological indicators for estimating the extent of functional disintegration of seed defences and the subsequent destabilisation of the intracellular architecture. Through this multivariate approach, we aim to bridge the gap between static biochemical profiling and dynamic physiological failure in long-term stored maize germplasm.

## 2. Results

To systematically evaluate the patterns of age-related change in physiological and biochemical markers during medium-term cold storage (CS), a comparative analysis is performed. Freshly regenerated seed samples harvested in 2022 serve as the control (CS0) group, providing a baseline against the original (CS1) samples harvested in 1985, which have undergone a 37-year cold-storage period.

### 2.1. The Seed Germination Potential

**Figure 1:**
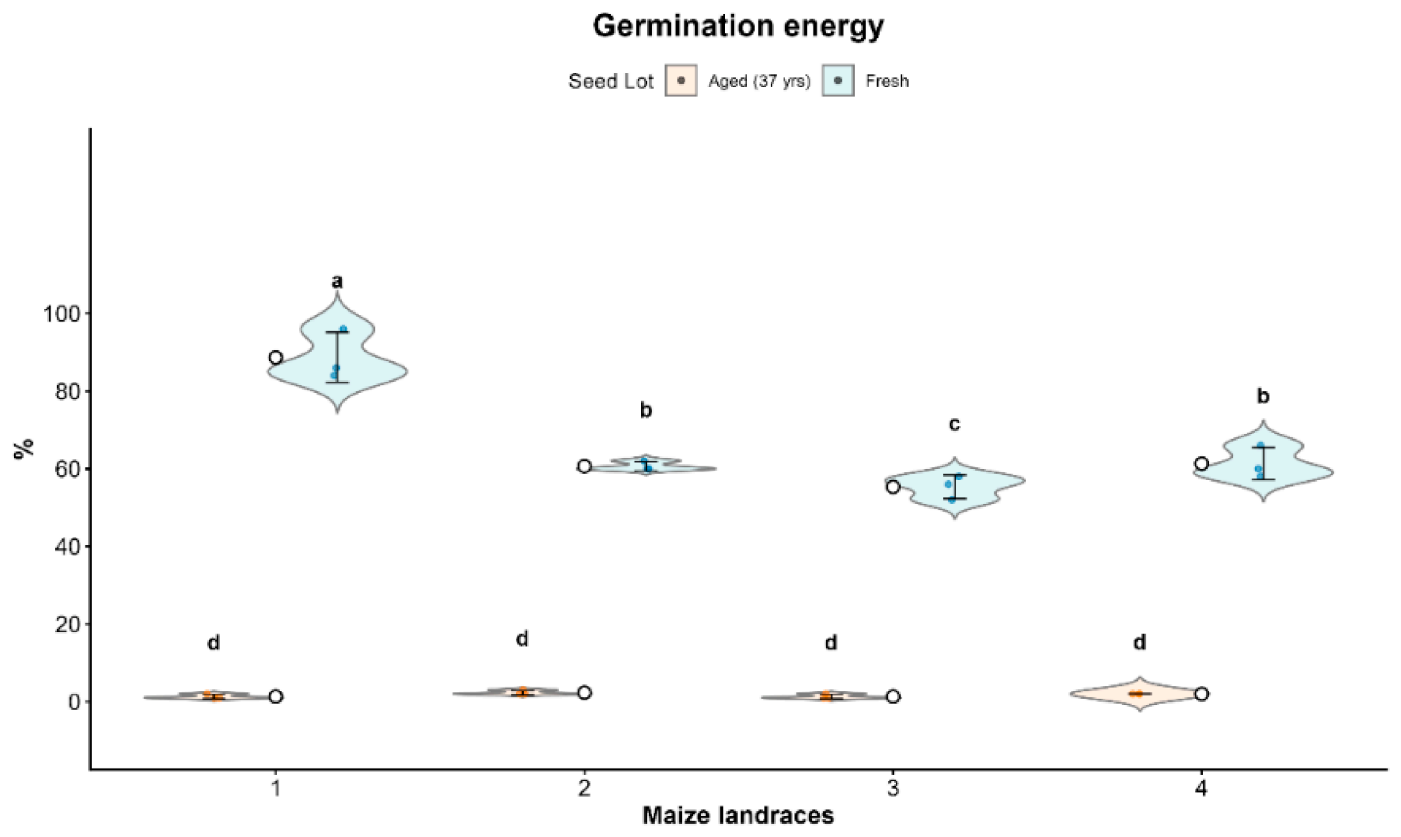
Germination Energy Divergence in Fresh and Aged Maize Landraces. GE (%) was evaluated for four maize landraces, each represented by an aged seed lots (CS1, 37 years of cold storage; odd-numbered samples 1, 3, 5, 7; orange violins) and a freshly regenerated control lot (CS0, harvested in 2022; even-numbered samples 2, 4, 6, 8; blue violins). The x-axis labels (1–4) denote the four distinct maize landraces. Different lowercase letters above the violin plots indicate significant differences (*p* ≤ 0.05) based on Fisher’s Least Significant Difference (LSD) test. The width of the violins represents the kernel density estimation of the data distribution, with individual replicates shown as internal points.

As a consequence of the natural ageing process during prolonged cold storage, the CS1 seed lots exhibit a uniform decline in germination potential. While the initial germination energy (GE) of the original samples exceeds 86%, after 37 years of medium-term cold storage, the germination energy across all four maize landraces—represented by the odd-numbered samples (1, 3, 5, and 7; orange violins)—collapses to near-zero levels (Figure 1). Conversely, the freshly regenerated control (CS0) seed samples, comprising the even-numbered samples (2, 4, 6, and 8; blue violins), maintain robust physiological vigour, though they display significant genetic variability. Sample 2 (landrace 1) achieved the highest performance at approximately 90%, while samples 4, 6, and 8 showed moderate levels ranging between 55% and 65%. Ultimately, the prolonged storage duration neutralises the initial performance differences observed in the fresh counterparts, resulting in a uniform “floor effect” where none of the aged seed lots retain the capacity for rapid germination (*p* ≤ 0.05).

**Figure 2:**
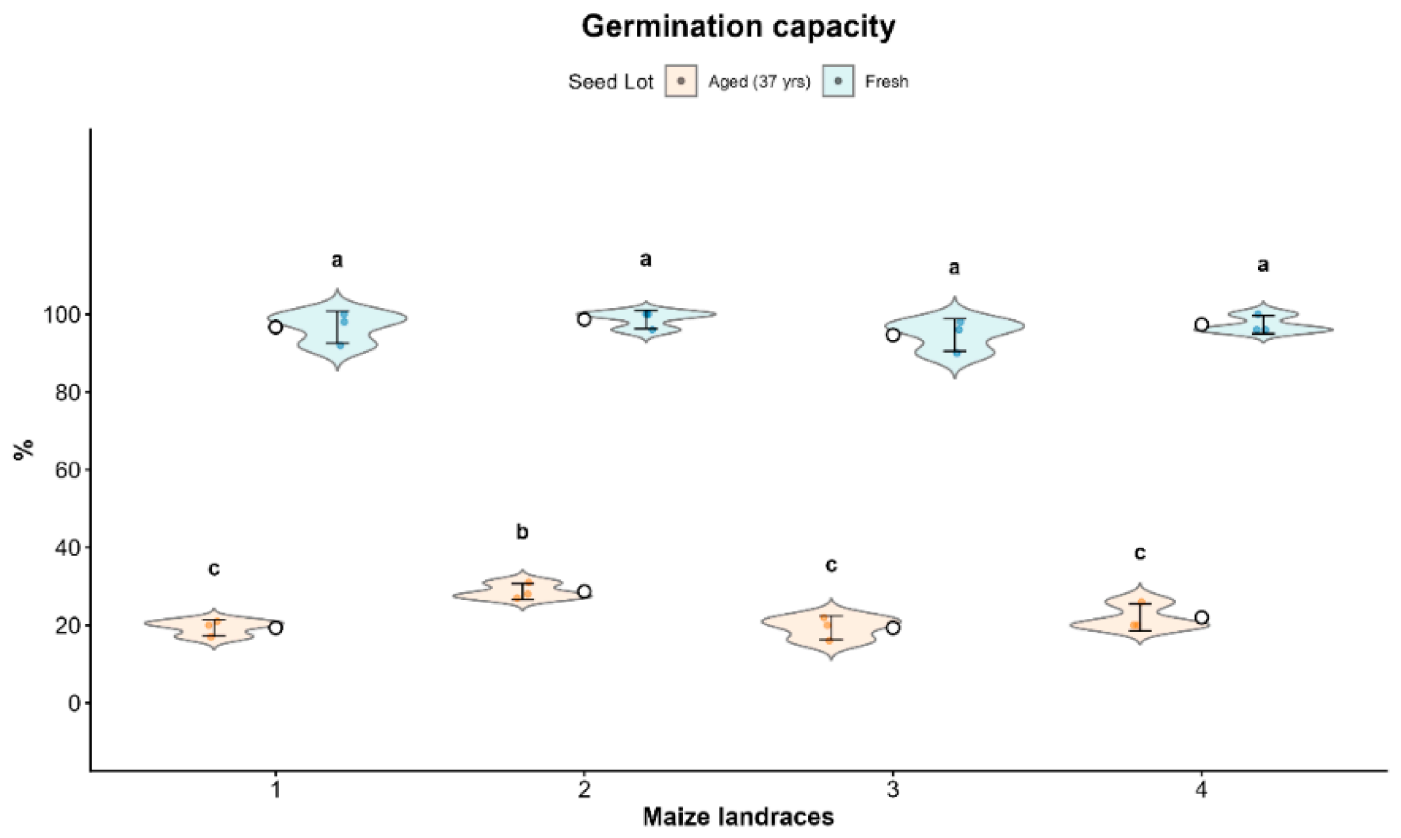
Germination Capacity Divergence in Fresh and Aged Maize Landraces. GC (%) was evaluated for four maize landraces (1–4), each represented by an aged seed lots (CS1, 37 years of cold storage; odd-numbered samples 1, 3, 5, 7; orange violins) and a freshly regenerated control lot (CS0, harvested in 2022; even-numbered samples 2, 4, 6, 8; blue violins). The x-axis labels (1–4) denote the four distinct maize landraces. Different lowercase letters above the violin plots indicate significant differences (*p* ≤ 0.05) based on Fisher’s Least Significant Difference (LSD) test. The width of the violins represents the kernel density estimation of the data distribution, with individual replicates shown as internal points.

While the GE (speed) is completely lost, the germination capacity (GC; total percentage) shows that a small fraction of the aged seeds are still viable, albeit significantly weakened. The initial germination capacity of the original CS1 samples exceeds 98%. However, after 37 years of medium-term cold storage, there is a significant average decline of approximately 77% in seed GC. The control samples (CS0; even-numbered samples 2, 4, 6, and 8; blue violins) displayed an average high GC of over 97%, maintaining a uniform level of high viability across all maize landraces. Conversely, the aged CS1 seed lots (odd-numbered samples 1, 3, 5, and 7; orange violins) exhibited a drastic reduction in total germination, with values plummeting to an average of roughly 21% (Figure 2). Among the aged lots, sample 3 (landrace 2) retains the highest residual capacity at approximately 28%, while samples 1, 5, and 7 performed significantly lower, clustering around 18–20%. These results indicate that while a small percentage of seeds remains viable after nearly four decades of cold storage, their physiological quality is severely compromised compared to the fresh controls.

### 2.2. Metabolic Drivers and Correlation Dynamics of Seed Germination Potential

While the absolute values of biochemical markers for these accessions were previously reported [19] and are summarized in Supplementary Tables S1–S4, the present study provides a high-resolution integrated re-analysis of their functional coordination over 37 years. To pinpoint the specific metabolic drivers of the physiological gap between physiological states, Pearson’s correlation coefficients (r) are calculated, contrasting the freshly regenerated baseline (CS0) against the aged seed lots (CS1).

**Figure 3:**
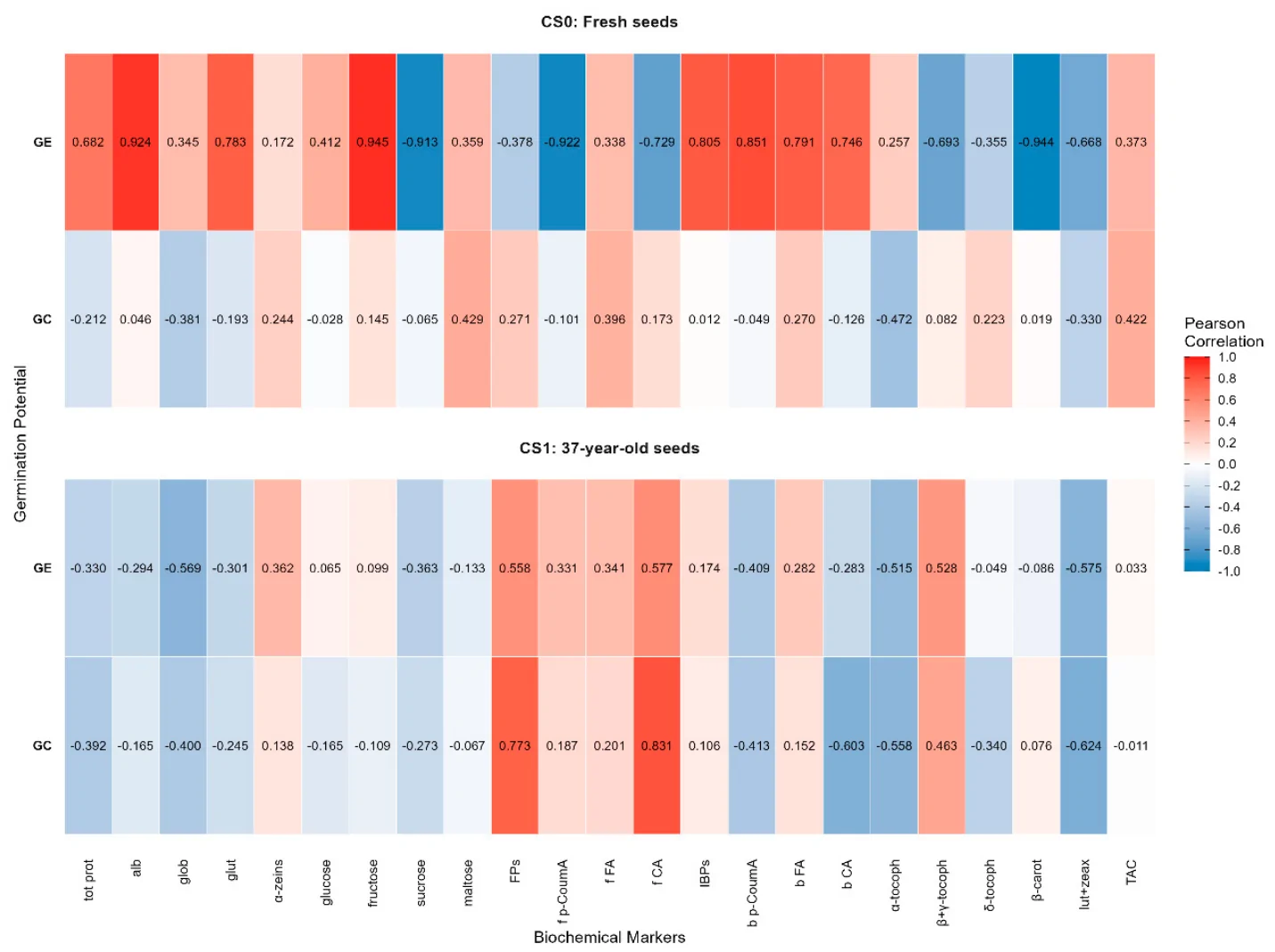
Germination Potential–Biochemical Marker Axis Reorganization. Heatmap illustrating the inter-relations between germination energy (GE) and germination capacity (GC) with biochemical parameters in freshly regenerated (CS0) and 37-year-old aged (CS1) maize seed lots. The colour scale represents Pearson’s correlation coefficients (r), ranging from blue (negative correlation, −1.0) to red (positive correlation, 1.0). Colour intensity denotes the strength of the association, while the divergence in colour patterns highlights the reorganization of biochemical roles during the ageing process. The critical values in a two-tailed test for a sample size of *n* = 12 are as follows: |r| ≥ 0.576 (at significance level *α* = 0.05), |r| ≥ 0.708 (*α* = 0.01), and |r| ≥ 0.823 (*α* = 0.001), respectively. Abbreviations: CS0/CS1—0 and 37 years of cold storage, respectively; GE—germination energy; GC—germination capacity; FPs—total soluble-free phenolics; IBPs—total insoluble-bound phenolics; f—soluble-free form; b—insoluble-bound form; *p*-CoumA—*p*-coumaric acid; FA—ferulic acid; CA—caffeic acid; TAC—Total Antioxidant Capacity.

The analysis reveals that the divergence in GE and GC is not stochastic but is associated with a profound reorganization of both primary and secondary metabolic networks. As shown in the correlation heatmap (Figure 3), the most significant indicators of viability loss are identified by the highly pronounced shift in correlation patterns between the CS0 and CS1 states.

In the high-vigour CS0 state, germination potential is characterised by a tightly coupled protein–sugar axis. This is evidenced by the highly significant positive correlations between GE and the total protein, i.e., albumin and glutelin fractions (r = 0.682, r = 0.924, and r = 0.783, respectively), alongside a near-total dependence on fructose availability (r = 0.945). Furthermore, a robust structural–antioxidant link is observed, where GE is positively anchored to the insoluble-bound phenolic pool—specifically total insoluble-bound phenolics (IBPs) (r = 0.805), *p*-coumaric acid (*p*-CoumA) (r = 0.851), ferulic acid (FA) (r = 0.791), and caffeic acid (CA) (r = 0.746). Conversely, the strong negative correlations with sucrose (r = −0.913), soluble-free *p*-CoumA (r = −0.922), and *β*-carotene (r = −0.944) suggest that in the CS0 state, high germination vigour is mechanically linked to the integrity of the protein matrix, specific hexose availability, and the stability of the insoluble-bound phenolic pool.

In stark contrast, the 37-year-old low-vigour CS1 seeds exhibit a widespread metabolic decoupling. The primary protein–sugar correlations that define vigour in fresh seeds effectively collapse, losing statistical relevance (*p* > 0.05). This indicates a systemic breakdown of the coordinated pathways required for rapid radicle protrusion. However, a distinct survival-oriented shift emerges in the aged state. In CS1 seeds, GC is no longer associated with primary metabolism but is instead anchored to the soluble-free phenolic pool, particularly total soluble-free phenolics (FPs) (r = 0.773) and free CA (r = 0.831). This free phenolic acid also serves as the sole remaining positive correlate for GE (r = 0.577), while a significant negative pressure on CS1 germination potential is exerted by lutein + zeaxanthin (r = −0.575 for GE, r = −0.624 for GC, respectively). These results demonstrate a fundamental transition: while fresh seeds rely on a growth-oriented biochemical profile and stabilized cell-wall structural integrity, long-term storage in the glassy state is characterized by the collapse of these functional associations, with residual viability in CS1 becoming coupled to the availability of FPs.

### 2.3. Systemic Reorganization of Biochemical Networks in Fresh and Aged Seeds

Natural ageing under prolonged cold storage induces a clear physiological divergence between CS0 and CS1 states, revealing a profound shift in multi-network coordination. Contrasting the high-resolution internal and intra-correlations of biochemical markers across both physiological states directly defines the relational reconfiguration of metabolic patterns and the emergence of a reconfigured biochemical coordination.

#### 2.3.1. Internal Correlations and Cross-Network Coordination of Biochemical Profiles

**Figure 4:**
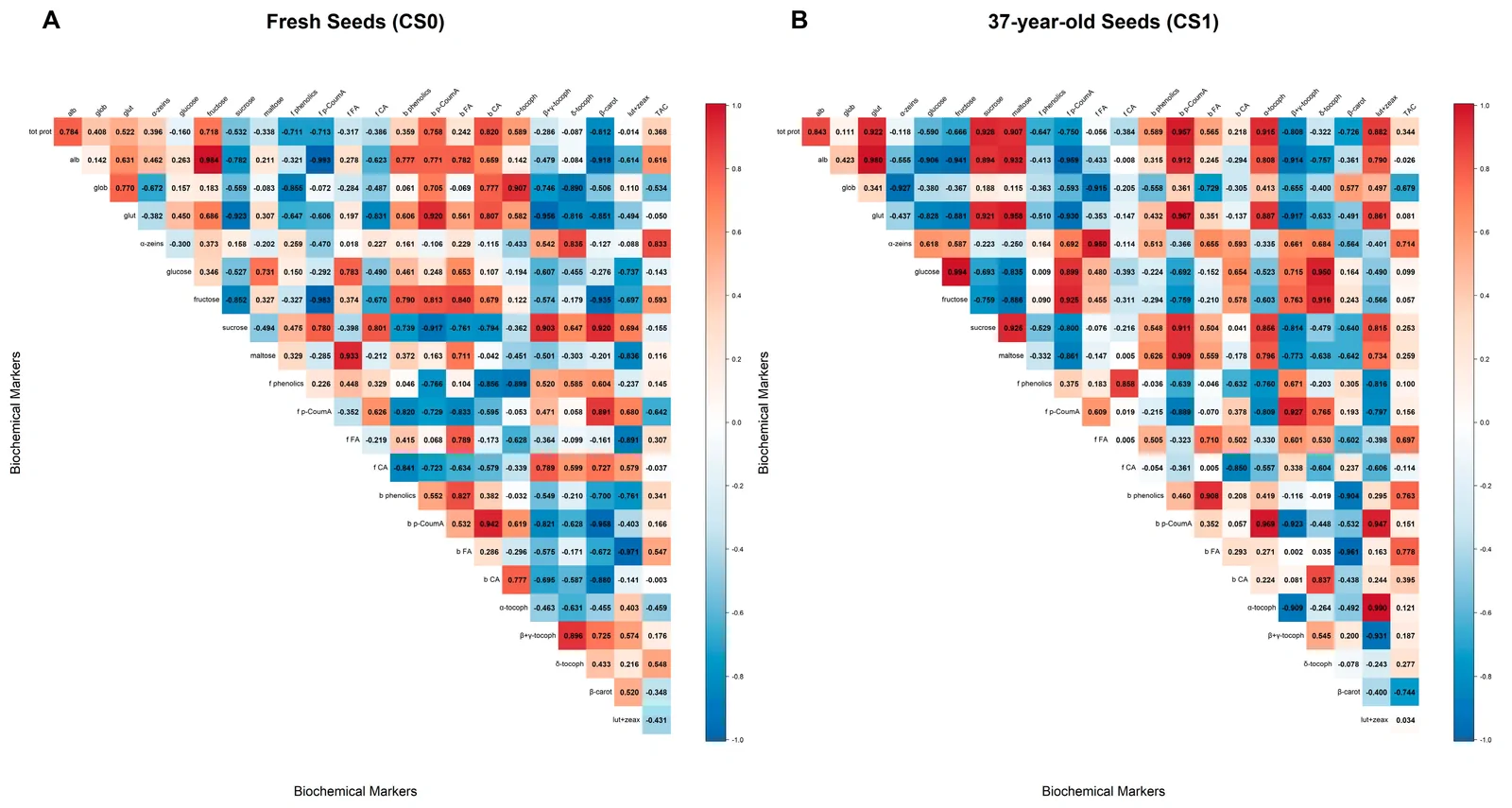
Multi-Network Biochemical Reorganisation and Coordination. Heatmap of Pearson’s correlation coefficients (r) illustrating the integrated internal correlations and cross-network intra-relations among proteomic, carbohydrate, phenolic and antioxidant matrices in (**A**) freshly regenerated (CS0) and (**B**) 37-year-old naturally aged (CS1) maize seed lots. The colour gradient illustrates the systemic redirection of biochemical coordination during decadal storage. The critical values of significance in a two-tailed test for a sample size of *n* = 12 are as follows: |r| ≥ 0.576 (*p* ≤ 0.05), |r| ≥ 0.708 (*p* ≤ 0.01), and |r| ≥ 0.823 (*p* ≤ 0.001), respectively.

Evaluation of the protein matrix reveals a non-uniform redistribution of fractions during decadal storage. The albumin pool displays a uniform depletion across all genotypes, strictly mirroring the progression of seed ageing (Table S1). In contrast, total protein and α-zeins contents decrease predominantly, while glutelin contents increase. Globulin trends remain genotype-specific, characterized by either stability or significant decrease depending on the sample. These divergent shifts directly alter the correlation patterns of the network observed in the transition from the high-vigour CS0 to the low-vigour CS1 state (Figure 4A,B). Within the proteomic matrix, CS0 is dominated by albumin-driven total protein status (r = 0.784), whereas the deteriorated CS1 state triggers profound internal consolidation, culminating in near-perfect albumin–glutelin coordination (r = 0.980) and strong negative decoupling of *α*-zeins. Across the protein–carbohydrate axis, the aged state shifts from hexose coordination toward a tight, positive disaccharide association, where albumins and glutelins achieve strong coordination with sucrose (r = 0.894 and r = 0.921, respectively) and maltose (r = 0.932 and r = 0.958, respectively). Furthermore, advanced ageing intensifies positive coordination between the albumins and glutelins pool and the bound phenolic cell wall architecture (particularly bound *p*-CoumA; (r > 0.910), whilst inducing systemic globulin decoupling from both carbohydrates and IBPs. Finally, the protein–antioxidant axis undergoes a profound redirection in the CS1 state, characterised by high-magnitude positive coordination of total protein, albumins, and glutelins with *α*-tocopherol (r > 0.800) and the lutein + zeaxanthin pool (r > 0.790), contrasted by profound intensification of negative coordination with *β*+*γ*-tocopherols.

Evaluation of the carbohydrate profile reveals a non-uniform redistribution of reducing and non-reducing sugars during decadal storage (Table S2). The maltose pool is unique in its uniform depletion across all evaluated genotypes, strictly mirroring the progression of seed ageing. In contrast, the hexose fractions exhibit divergent predominant trajectories; glucose displays a predominant decrease, whereas fructose exhibits a predominant content increase. Variations in sucrose content remain strictly genotype-specific. This restructuring drives sharp pattern inversions across all molecular axes during the CS0-to-CS1 transition (Figure 4A,B). The CS1 monosaccharide matrix enters near-perfect coordination (r = 0.994 between glucose and fructose), hexose–maltose dynamics invert to robust negative coordination (r = −0.835 for glucose; r = −0.886 for fructose), and the sucrose–maltose relationship reverses from negative to highly positive (r = 0.925). Across the carbohydrate–phenolic axis, CS1 monosaccharides closely track free *p*-CoumA (glucose: r = 0.899; fructose: r = 0.925), whilst sucrose and maltose achieve powerful positive integration with bound *p*-CoumA (r = 0.911 and r = 0.909, respectively). Finally, the carbohydrate–antioxidant axis reorients radically; CS1 monosaccharides develop near-perfect coordination with *δ*-tocopherol (glucose: r = 0.950; fructose: r = 0.916), whilst sucrose and maltose establish robust positive links with *α*-tocopherol (r = 0.856 and r = 0.796) and lutein + zeaxanthin (r = 0.815 and r = 0.734), contrasted by deep sucrose–*β*+*γ*-tocopherol antagonism (r = −0.814).

Evaluation of the phenolic profile reveals its complex redistribution during decadal storage (Table S3a,b). Both the total FPs and the total IBPs contents exhibit predominant stability across the evaluated genotypes. The specific phenolic acids follow more defined, independent trajectories. While FA displays a uniform increase in both its free and bound forms, *p*-CoumA exhibits a predominant increase in the free form contrasting with a predominant decrease in the bound form. Variations in CA content remain strictly genotype-specific, with these fluctuations being more pronounced in the bound form. During the CS0-to-CS1 transition (Figure 4A,B), the internal phenolic network reconfigures as strong CS0 negative links—total FPs vs. bound CA (r = −0.856) and total IBPs vs. free CA (r = −0.841)—invert in CS1 to powerful positive total FPs-free CA coordination (r = 0.858), alongside intensified free-bound antagonism for CA (r = −0.850) and *p*-CoumA (r = −0.889). Across the phenolic–antioxidant axis, the extreme CS0 inverse bound FA–lutein + zeaxanthin alignment (r = −0.971) shifts in CS1 to extreme positive couplings of bound *p*-CoumA with α-tocopherol (r = 0.969) and lutein + zeaxanthin (r = 0.947), and free *p*-CoumA with *β*+*γ*-tocopherol (r = 0.927). Finally, TAC redirects from its negative CS0 link with free *p*-CoumA (r = −0.642) to track bound FA (r = 0.778), total IBPs (r = 0.763), and free FA (r = 0.697) in the aged matrix.

Seed ageing induces a systematic depletion of the protective lipophilic antioxidant shield, resulting in a predominant decrease in all tocopherol isomers, and a uniform pattern of decline in the carotenoid pool, with the TAC exhibiting absolute stability (Table S4). This decline drives a systematic reorientation of the network architecture during the CS0-to-CS1 transition (Figure 4A,B). In the high-vigour CS0 state, the antioxidant pool is characterised by tight internal coordination, highlighted by a robust positive link between *β*+*γ*-tocopherols and *δ*-tocopherol (r = 0.896). Upon transitioning to the low-vigour CS1 state, these patterns undergo substantial redirection; *α*-tocopherol develops an intensified negative correlation with *β*+*γ*-tocopherols (r = −0.909) alongside a near-perfect positive coordination with lutein + zeaxanthin (r = 0.990). Conversely, the association between *β*+*γ*-tocopherols and lutein + zeaxanthin radically reverses to an extreme negative correlation (r = −0.931), whilst TAC develops a strong inverse correlation with *β*-carotene (r = −0.744) in the aged matrix.

#### 2.3.2. Multivariate Analysis of Physiological and Biochemical Divergence

Individual Principal Component Analysis (PCA) biplots are constructed to resolve how distinct functional domains define seed viability across the two physiological states, effectively mapping the transitions from a high- (CS0) to a low-vigour (CS1) state.

For the proteomic matrix, the total variance explained by the first two components reaches 80.48%, demonstrating a clear separation of distinct vigour states (Figure S1, Tables S5 and S9a). The primary vigour axis (PC1, 49.09% of variance) is defined by high positive loadings for GE and GC, which are strongly coordinated with the total protein and albumins, identifying this storage protein fraction as the primary biochemical anchor for physiological potential. Conversely, the structural redirection of the matrix on PC2 (31.39% of variance) is driven by a sharp pattern inversion where α-zeins exhibit a powerful negative loading, standing in direct opposition to the glutelin and globulin fractions in the low-vigour (CS1) state.

Regarding the carbohydrate profile, a substantial 86.66% of the total variance is captured by the first two components, highlighting the profound physiological and biochemical divergence on PC1 (48.62% of variance) where exceptionally high positive loadings for GE and GC coordinate with maltose and glucose (Figure S2, Tables S6 and S9b). Conversely, a biophysical stability axis (PC2, 38.04% of variance) is driven by a sharp pattern inversion between the non-reducing and reducing sugar pools, where the powerful positive loading of the non-reducing sugar pool stands in direct vertical opposition to the strong negative alignments of the reducing sugar pool. The PCA biplot (Figure S2) visually captures this profound systemic shift following prolonged cold storage, where the high-vigour (CS0) seeds form a cohesive cluster defined by a robust non-reducing sugar balance, whereas the low-vigour (CS1) samples shift in opposition to the germination potential vectors under the influence of hexose accumulation and unique genetic backgrounds.

In the case of the phenolic matrix, the two primary components resolve 72.51% of the total variance, isolating the high-vigour characteristics along the primary vigour axis (PC1, 38.42% of variance) through exceptionally high positive loadings for GE and GC, which stand in direct horizontal opposition to the accumulated free phenolic pool (Figure S3, Tables S7 and S9c). Conversely, a chemical architecture axis (PC2, 34.08% of variance) is driven by a sharp pattern inversion between the bound and free phenolic pools, where the powerful positive loadings of the bound phenolic pool stand in direct vertical opposition to the strong negative alignments of the FPs pool. The PCA biplot (Figure S3) visually captures this profound systemic shift following 37 years of cold storage, where the high-vigour (CS0) seed lots form a cohesive cluster aligned with high germination potential, whereas the low-vigour (CS1) samples shift in opposition to these vectors under the influence of free phenolic mobilisation, bound fraction deterioration, and unique genetic backgrounds.

Finally, the component resolution of the antioxidant pool accounts for 77.87% of the total variance, mapping the distinct functional dimensions of the lipophilic defence system (Figure S4, Tables S8 and S9d). The primary vigour axis (PC1, 54.22% of variance) is established through exceptionally high positive loadings for GE and GC coordinated with the accumulated carotenoid pool. Conversely, PC2 (23.65% of variance) functions as an antioxidant architecture axis driven by a sharp pattern inversion where the powerful negative loading of *α*-tocopherol stands in direct vertical opposition to the strong positive alignments of the remaining tocopherol isomers. The PCA biplot (Figure S4) visually captures this profound systemic shift following decadal cold storage, where the high-vigour (CS0) seeds form a cohesive cluster aligned with high germination potential, whereas the low-vigour (CS1) samples shift in opposition to these vectors under the influence of carotenoid pool exhaustion, accession-specific tocopherol redistribution, and unique genetic backgrounds.

An integrated Principal Component Analysis (PCA) is conducted to resolve the multi-network coordination of biochemical and physiological variables governing seed viability and deterioration. This multivariate synthesis reveals four principal components with Eigenvalues greater than 1, which collectively account for 88.85% of the total variance (Table 1).

The first principal component (PC1, 31.49% of variance) functions as the primary axis defining physiological vigour, effectively separating high germination potential from subsequent degradation shifts (Table 1 and Table S9e). This dimension is characterised by exceptionally high positive loadings for GE and GC, which are strongly coordinated with albumins, total protein, and bound *p*-CoumA. Furthermore, the lipophilic antioxidants *α*-tocopherol and the lutein + zeaxanthin pool align strongly on this positive plane, acting as primary indicators of the high-vigour state. Conversely, the transition to the low-vigour state along this axis is marked by strong negative alignments for free *p*-CoumA, free FA, and fructose, illustrating that viability loss directly coincides with the comprehensive hydrolytic collapse of cellular and endosperm structures, marked by the release of hydroxycinnamic monomeric debris from cell wall polymers.

Resolution of the second principal component (PC2, 26.96% of variance) uncovers a critical regulatory axis driven by the reorganisation of storage reserves and the differential mobilisation of the lipophilic pool (Table 1 and Table S9e). This metabolic dimension is dominated by a pronounced inverse relationship between the storage proteome and specific tocopherol isomers. Notably, *β*+*γ*-tocopherols and *δ*-tocopherol display exceptionally strong positive alignments, standing in direct vertical opposition to the retention integrity of the glutelin fraction. Significant positive weight for *β*-carotene further anchors this positive plane, reflecting genotype-specific baselines of endosperm preservation within the high-vigour state. Ultimately, the total weighted contribution computed across this multivariate model identifies glutelins and *β*+*γ*-tocopherols as the most influential variables across the evaluated physiological states.

**Figure 5:**
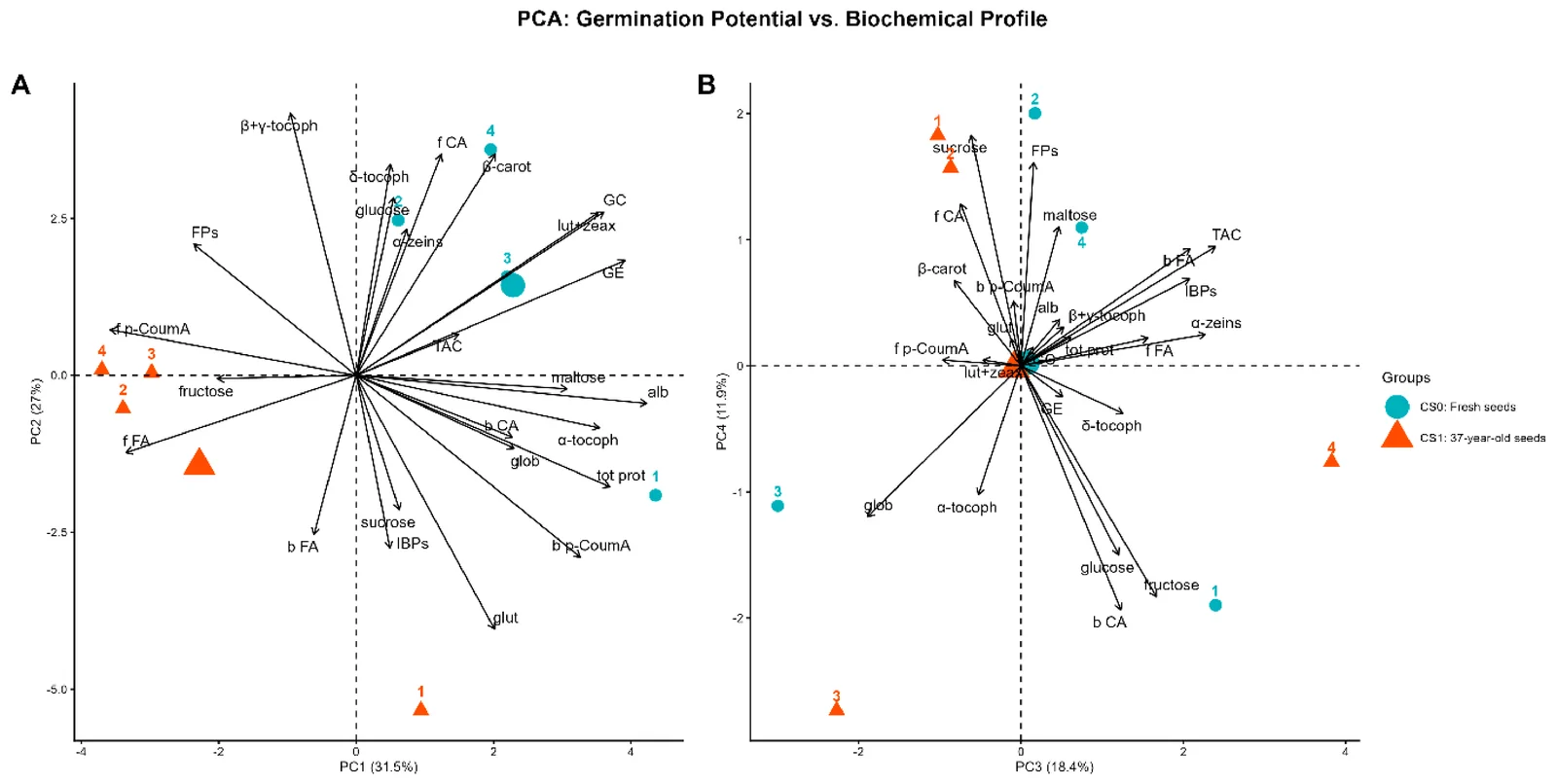
PCA biplot illustrating the coordination between germination potential and the integrated biochemical profile in (**A**) fresh (CS0; blue circles) and (**B**) 37-year-old (CS1; red triangles) maize seed lots. Vectors indicate factor loadings for the integration of physiological and biochemical variables; numbers (1–4) identify individual maize landrace accessions. Abbreviations: GE–Germination energy; GC–Germination capacity; TAC–Total Antioxidant Capacity.

The integrated biplot space for PC1 vs. PC2 (Figure 5A) visually maps these multi-network transitions, sorting the germplasm according to physiological fitness through a clear spatial separation between distinct vigour states. The upper-right quadrant defines the active antioxidant and pigment-mediated protection domain within the fresh high-vigour (CS0) state; within this zone, CS0 seed lot 3 exhibits the highest physiological fitness, mapping directly at the apex of the longest and most critical vectors for GE, GC, TAC, and the lutein + zeaxanthin pool, supported by the CS0 seed lot 4 along the *β*-carotene axis and the CS0 seed lot 2 near the α-zeins, glucose, and *δ*-tocopherol vectors. Conversely, the lower-right quadrant represents the primary metabolism and storage proteome preservation domain, where the fresh CS0 seed lot 1 anchors its metabolic profile along exceptionally long vectors for total protein, albumins, and globulins coordinated with maltose and bound *p*-CoumA. Following 37 years of cold storage, the prolonged aged low-vigour (CS1) state seed lots undergo dramatic, divergent redirections. The transition toward seed ageing shifts the majority of these samples into the left-hand quadrants, driven by a powerful pattern inversion relative to the germination vectors. Within this plane, the upper-left quadrant functions as the stress-induced phenolic and tocopherol mobilisation domain, where the aged CS1 seed lots 3 and 4 align with the prominent accumulation vectors of free *p*-CoumA and the total FPs pool. Conversely, the lower-left quadrant represents the terminal matrix collapse and lipid degradation domain, which directly drives the horizontal migration of the aged CS1 seed lot 2 along the maximum-length vector for free FA, running parallel to fructose and free ferulic acid. Distinctly, the aged CS1 seed lot 1 bypasses this comprehensive lateral collapse by retaining its position within the lower margin of the right-hand plane, maintaining isolated physiological resilience through a direct alignment with the vertical storage vectors of sucrose, total IBPs, and glutelins.

Transitioning to the third principal component (PC3, 18.44% of variance), the multi-network alignment captures the structural preservation limits of hydrophobic α-zeins in tandem with the residual reductive capacity of the seed tissue (Table 1). On this axis, TAC strongly dominates the positive plane, serving as a comprehensive marker for the remaining scavenging potential within the aged germplasm. This antioxidant parameter runs intrinsically parallel to high positive projections for *α*-zeins, bound FA, and the total IBPs pool, demonstrating a tight coordination between the preservation of specialised protein body organelles and the retention of the bound cell wall lattice.

The architectural layout of the fourth principal component (PC4, 11.95% of variance) reveals the terminal disruption of cellular matrix compartmentation (Table 1). This final dimension highlights a pronounced inverse configuration where sucrose develops a strong positive loading in direct coordinate opposition to the negative alignments of bound CA, fructose, and glucose. This geometry effectively captures the biophysical boundary where the release of IBPs from the cell wall scaffold co-occurs with the critical relaxation and structural breakdown of the carbohydrate glass.

The multidimensional map in Biplot B (PC3 vs. PC4) establishes a refined separation based on localised reserve protection and active structural maintenance (Figure 5B). The upper-right quadrant defines the active structural and antioxidant stability domain of the seeds, characterised by an integrated network of TAC, total FPs, and *β*+*γ*-tocopherols working in tandem with total IBPs, bound FA, *α*-zeins, albumins, total protein, GC, and maltose; within this core framework, the fresh high-vigour CS0 seed lots 2 and 4 display a maximum-capacity alignment along this integrated protective network, directly driven by the long vector of TAC. Transitioning to the upper-left quadrant, the biplot isolates the passive, carbohydrate-mediated glass stabilisation domain, which is dominated by sucrose, glutelins, free CA, free *p*-CoumA, bound *p*-CoumA, *β*-carotene, and the lutein + zeaxanthin pool; this domain uniquely captures the physiological resilience of the aged low-vigour CS1 seed lots 1 and 2, which maintains matrix integrity through the co-preservation of its carbohydrate and storage proteome. Conversely, the lower-right quadrant of Biplot B highlights the structural matrix and hexose turnover domain, marked by the coordinate alignment of glucose, fructose, *δ*-tocopherol, and bound CA. This domain resolves a highly divergent physiological character, anchoring the fresh CS0 seed lot 1 along the vertical axes of accelerated hexose mobilisation, while capturing the spontaneous release of free hexoses resulting from the degradation and structural failure of cell wall polymers in the aged CS1 seed lot 4. Finally, a shift toward the lower-left quadrant resolves the tissue-specific embryo and lipid protection domain, defined by a distinct coordination between globulins and *α*-tocopherol. This domain highlights a profound age-induced divergence, where the fresh CS0 seed lot 3 remains positioned closer to the centre, whereas the aged CS1 seed lot 3 is markedly displaced deep along these protective vectors, reflecting a specialised, non-germinative survival or residual defence response resulting from decadal ageing.

## 3. Discussion

### 3.1. Biological Limits and Germination Decay Kinetics in an Active Collection

The 37-year-term cold storage of the four evaluated maize landraces results in a near-total collapse of germination energy (initial GE > 86% vs. current GE ≈ 2%) alongside a severe attenuation of germination capacity to approximately 21%. While seeds evolve specialised mechanisms to withstand environmental stress, they naturally undergo an inevitable physiological ageing process that progressively compromises subcellular and biochemical integrity [14]. The stark divergence between GE and GC directly quantifies the terminal energetic exhaustion of these low-vigour aged seed lots, providing significant diagnostic value that complements our previous evidence of declining seedling growth performance [19].

This pronounced decay trajectory far exceeds the expected two-decade security window for medium-term storage, highlighting the physiological risks of extending storage within an active collection framework (+4 °C) beyond its intended operational lifespan [4,20]. Similar pronounced viability losses (down to 35%) have been documented for wheat and barley stored under comparable medium-term conditions [17], confirming a shared cross-species transition from a homeostatic viability plateau to a sharp decadal viability decline. However, despite the near-total exhaustion of vigour, the evaluated aged maize seed lots retain a residual germination capacity of ~21%, likely supported by optimal exogenous laboratory conditions that temporarily mask the true extent of intrinsic physiological degradation [21].

This transition from slow, reparable damage to an irreversible high-mortality phase is fundamentally dictated by the thermodynamic constraints of storage and the exhaustion of endogenous antioxidant defences against reactive oxygen species (ROS) [4,22]. The preservation of the residual 21% viability is tightly linked to the stabilising effect of the intracellular glassy matrix [23,24]. However, the persistence of a slow molecular diffusion rate over nearly four decades eventually allows matrix viscosity to reach a critical threshold, driving the observed biochemical degradation [24,25].

Furthermore, the operational requirements of active collections—specifically periodic exposure to transient ambient humidity shifts during frequent sample retrievals—likely accelerate this deterioration [26]. These cumulative hygroscopic events lower cytoplasmic viscosity and induce structural relaxation of the glassy matrix, hastening downstream biochemical degradation pathways and culminating in the complete exhaustion of repair-and-defence strategies–the observed “floor effect” in germination energy and the steep drop in overall seed viability.

### 3.2. Redirection of the Biochemical Landscape: From Metabolic Efficiency to Matrix Relaxation

A high-resolution integrated analysis of biochemical coordination reveals that the physiological divergence between the high-vigour and low-vigour states is defined by a profound reconfiguration of the seed’s integrated biochemical profile. In the high-vigour state, GE serves as a sensitive indicator of immediate metabolic efficiency [7], demonstrating a tight physiological coupling with the availability of total proteins, albumins and glutelins. This alignment identifies a prime physiological configuration where the velocity of radicle protrusion relies on an accelerated hexose turnover, quantitatively supported by the strong dependence on fructose availability and the concurrent rapid depletion of the sucrose pool. This uncoupling indicates that in these high-vigour seeds, immediate germinative energy is achieved through the intense enzymatic activation of early carbohydrate mobilisation and respiratory pathways. This process draws directly from a functional metabolic architecture where the rapid utilisation of the pre-assembled functional proteome and available hexose intermediates serves to metabolically fuel the early respiratory pathways and cellular repair networks required to sustain rapid germination [23,27]. In contrast, the lack of significant coordination between germination capacity and these biochemical markers in fresh seeds indicates that germination capacity represents a broader measure of seed viability, largely independent of the immediate kinetics of the metabolic restart; in high-vigour seeds, the capacity to eventually germinate remains a stable baseline not yet constrained by the quantitative depletion of reserves or structural decay [28]. This physiological coordination is physically anchored by a robust structural–antioxidant (i.e., insoluble-bound phenolic) pool, which likely contributes to maintaining the structural stability of the intracellular glass matrix by restricting molecular mobility and preserving the primary enzymatic and metabolic drivers of vigour [24,29]. These key biochemical fractions emerge as potential constitutive indicators of storability.

However, 37 years of cold storage result in a systemic redirection, wherein the primary correlations defining fresh seed vigour effectively collapse. The loss of statistical relevance for the protein–sugar axis in the aged state indicates a terminal breakdown of the coordinated metabolic pathways required for successful germination. This redirection is a direct physiological consequence consistent with the theory of cytoplasmic glass-state decay, suggesting that the progressive molecular relaxation of the glass over decadal storage permits a critical increase in localised cytoplasmic molecular mobility. This internal shift drives the systemic decoupling of previously restricted metabolic components, accelerating deleterious, non-enzymatic reactions and causing irreversible macromolecular damage during long-term cold storage [6,25]. Moreover, a compensatory redirection emerges, wherein germination potential is no longer linked to the systemic antioxidant capacity, but is instead associated with specific solubilised intermediates arising from cellular degradation. The shift in germination capacity toward a highly significant positive correlation with the total soluble-free phenolics and free caffeic acid represents a terminal shift in the biochemical landscape of the low-vigour seed. However, given the negligible free caffeic acid content in the aged accessions [19], this pronounced coordination likely functions as an indirect proxy indicator for structural cell wall breakdown and intracellular matrix relaxation, offering a testable hypothesis for glass destabilisation rather than reflecting an active, functional defensive strategy.

Furthermore, the stability of the total soluble-free phenolics content over decadal cold storage, coupled with its significant correlation with GC, indirectly suggests that the molecular relaxation of the glassy state has fundamentally altered the molecular accessibility of these compounds, rendering this fraction a physiological indicator for the degree of matrix relaxation as the cell wall lattice disintegrates [6,25]. Moreover, a profound decoupling of TAC from germination potential in the aged state implies that although a general basal scavenging potential persists [19], it is physically isolated or kinetically restricted, and thus no longer integrated into the physiological requirements of the active germination process [30]. In this context, the low-vigour seeds are increasingly pressured by the critical exhaustion of the lipophilic lutein + zeaxanthin pool. This depletion represents a tangible, pernicious loss of lipid-soluble plastidial protection that the re-exposed structural phenolic fraction is completely insufficient to compensate for, leaving cellular membranes entirely vulnerable to lethal oxidative deregulation and lipid peroxidation upon subsequent imbibition [31]. Consequently, the exhaustion of the carotenoid pool signifies a collapse of the cell’s redox homeostasis; without this integrated defence, the seed is unable to buffer the reactive oxygen species generated during rehydration, rendering the transition from dry storage to active metabolism physiologically impossible [32,33].

### 3.3. Coordination and Functional Disruption of Biochemical Networks: Mapping the Systemic Collapse of Intracellular Architecture

#### 3.3.1. Intra-Matrix Reconfiguration and the Physiological Erosion of Germination Potential

This terminal vulnerability reflects a systemic spatial re-ordering of macromolecular components, shifting the intracellular environment from a state of functionally partitioned metabolic autonomy to a structural and biochemical entanglement that incapacitates germinative activation.

The transition from high vigour to physiological exhaustion entails a structural proteome inversion. High-vigour seeds exhibit distinct compartmentalisation; coordinated globulin and glutelin pools may reflect an integrated storage system poised for rapid breakdown, coinciding with fructose availability for immediate metabolic restart [34]. Conversely, advanced ageing appears to induce macromolecular cross-linking and physical entrapment of storage fractions (albumins and glutelins) within the intracellular matrix [35]. As the intracellular glass matrix undergoes decadal molecular relaxation, specific storage proteins develop a near-perfect alignment with sucrose. This behaviour is broadly compatible with theoretical expectations of glass-state decay, where the sucrose glass potentially functions as a driver of macromolecular confinement [36], thereby intimating a restriction of kinetic fluidity [5,25] and enzymatic activation required for metabolic recovery [14].

This immobilisation extends to the antioxidant landscape. In high-vigour seeds, tocopherol–glutelin coordination presumably ensures lipophilic membrane protection [37], whereas aged seeds show patterns indicative of comprehensive *α*-tocopherol and carotenoid alignment with aggregated proteins, rendering these pigments functionally sequestered and potentially shifting diffusion-limited scavenging toward kinetic arrest [38,39]. The hydrophobic *α*-zein fraction remains isolated within protein bodies [40,41], yet its convergence with reducing hexoses and mobile free phenolic acids (*p*-CoumA, FA) points toward a non-functional association driven by proximity as cell-wall lattices disintegrate [42]. Concurrently, the consistent partitioning of TAC into the *α*-zein matrix likely mirrors localised chemical debris and glycation intermediates rather than active defence [43,44], thereby constituting a methodological limitation that warrants component-level validation. Ultimately, this transition from substrate-specific coordination to systemic proteomic immobilisation corresponds to the final locking of protective and nutritional systems into a rigid state that may irrevocably compromise redox homeostasis [33,45], ultimately aligning with the cessation of radicle protrusion.

The protein composition of the quiescent seed aligns directly with early post-germinative growth dynamics [19]. In a high-vigour state, the coordinated accumulation and structural integrity of total proteins and the glutelin fraction support active seedling growth by serving as important metabolic substrates for nitrogen mobilisation. Conversely, during prolonged ageing, the ROS-mediated carbonylation and macromolecular cross-linking impair glutelin functionality despite stable total protein content. Consequently, the accumulation of these damaged protein aggregates limits subsequent reserve utilisation and hinders dry weight accumulation, directly accounting for the downstream reductions in seedling performance and developmental plasticity established in our previous study [19].

Shifts in internal carbohydrate coordination are broadly compatible with glass-state decay theory, marking a transition from metabolic fluidity in fresh seeds to a putative loss of compartmental barriers during advanced ageing. In high-vigour seeds, the hexose pool maintains metabolic autonomy, suggesting sustained accessibility for glycolysis upon imbibition. Conversely, advanced ageing induces a near-perfect hexose coordination, which may reflect mobility constrained by the dense proteomic–sugar matrix and potentially creating a kinetic bottleneck that triggers rapid ATP starvation [46]. Furthermore, the inversion of glucose–maltose coordination [27,47] is consistent with hexose sequestration via non-enzymatic glycosylation, thereby intimating suppressed substrate fluidity [6,48].

In the phenolic matrix, sucrose maintains metabolic fluidity through exclusion from bound phenolics, whereas the aged state points toward a potential loss of compartmental barriers. The diagnostic marker of this redirection is sucrose immobilisation, whose near-perfect coordination with bound *p*-CoumA [49,50] is consistent with entrapment within the degrading cell wall matrix [25]. This persistent molecular entrapment likely creates a severe transport blockade, presumably restricting symplastic or apoplastic translocation to the embryonic axis [51]. Concurrently, the disintegration of the maltose–ferulic acid coordination corresponds to a physical immobilisation that may permanently obstruct the molecular fluidity required for germination [52,53].

The dissolution of the carbohydrate–antioxidant boundary further defines the biological limit of stored germplasm. In high-vigour seeds, weak coordination of reducing hexoses with antioxidants presumably prevents the nucleophilic Maillard cascade [43,54,55]. However, aged seeds exhibit massive coordination inversions: sucrose immobilisation—tightly aligned with *α*-tocopherol alongside *β*-carotene depletion—reflects patterns indicative of compromised free radical defence [39]. Moreover, the forced coordination of reducing hexoses with lipophilic antioxidants is compatible with desiccation-driven membrane degradation, potentially entrapping tocopherols within a glycated scaffold and restricting radical-scavenging mobility [6,56,57]. Ultimately, the functional disintegration of TAC coordination likely mirrors matrix stabilisation failure, thereby precipitating the collapse of cellular redox homeostasis [6].

The structural organisation of the carbohydrate matrix demonstrates a clear physiological linkage with initial seedling development, extending the homeostatic frameworks established in our previous study [19]. In a high-vigour state, metabolic autonomy of the hexose pool is reflected by strong positive associations of fructose with root and shoot elongation, confirming its role as an immediate respiratory substrate, despite a presumable fructose over-reliance that limits sustained dry matter accumulation. Conversely, the inherently lower sucrose content of these maize landraces (i.e., genotypes with standard quality grain) exhibits an inverse relationship with initial elongation, yet strongly supports dry biomass accumulation. However, during prolonged ageing, this regulated carbohydrate dynamic completely collapses. The massive accumulation of non-mobilised fructose in low-vigour old seeds ceases to serve as energetic fuel, instead driving non-enzymatic glycation and oxidative degradation that impair seedling growth. Simultaneously, as sucrose becomes biochemically immobilised and entrapped within the degrading cytoplasmic matrix, its positive coordination with biomass accumulation disappears, while its relationship with shoot length undergoes a major pattern inversion to a strong positive correlation. This structural redirection demonstrates that the biophysical confinement of the transport sugar directly limits subsequent reserve utilisation, accounting directly for a shift where initial shoot elongation is maintained at the expense of biomass accumulation during early seedling establishment [19].

The coordination within the internal phenolic compartment and the phenolic–antioxidant network serves as a fundamental indicator of seed performance, reflecting the structural integrity of the cell wall lattice and chemical compartmentation. In a high-vigour state, the internal phenolic matrix is anchored by coordination between bound *p*-CoumA and bound FA, implying an organised structural network integrated into the cell wall via complex ferulate cross-linking [58]. This state features strict metabolic partitioning between total FPs and insoluble bound fractions (*p*-CoumA, CA), ensuring a protective antioxidant array for the embryo [59,60]. Within the wider network, phenolic and antioxidant pools maintain strict exclusion boundaries through spatial isolation of total FPs and bound FA away from *α*-tocopherol and carotenoids, preserving radical-scavenging efficiency within membrane bilayers and the vitrified cytoplasm [61,62]. Concurrently, positive coordination between free *p*-CoumA and carotenoids indicates a regulated response within the mobile phase, maintaining lipophilic integrity during quiescence [24].

Conversely, advanced ageing points toward a potential loss of compartmental barriers and biophysical destabilisation. A primary diagnostic marker is the pattern inversion between free *p*-CoumA and free FA, shifting into tight positive coordination alongside the disintegration of the insoluble bound phenolic network during decadal molecular relaxation of the vitreous matrix [25]. Concurrently, macromolecular entrapment intensifies within the bound matrix (total IBPs and bound FA). As cellular membranes fail, previously isolated fractions undergo forced intermixing: the bound *p*-CoumA and *α*-tocopherol relationship intensifies into a coupled coordination. This phenomenon likely stems from membrane permeability loss and progressive macromolecular crowding [38], potentially driving phenolic and antioxidant immobilisation via non-specific matrix co-aggregation, rendering them biologically unavailable to counteract oxidative damage [24]. Ultimately, the redirection of TAC and total IBP coordination is consistent with matrix stabilisation failure, where the restricted molecular mobility compromises defence and precipitates the loss of cellular redox homeostasis upon imbibition [33,63].

The biochemical constitution of the internal phenolic framework aligns closely with subsequent seedling growth kinetics, consistent with the developmental trade-offs identified in our previous study [19]. In a high-vigour state, the active metabolic breaking of ester bonds within the cell wall lattice releases bound phenolic acids to facilitate wall loosening and cell expansion, promoting rapid root and shoot elongation; however, this intense metabolic demand accelerates reserve consumption, resulting in a distinct trade-off where initial growth occurs at the expense of dry biomass accumulation. Conversely, during prolonged ageing, this highly regulated structural control is permanently lost. The biophysical entrapment and altered dynamics of bound FA enhance non-specific oxidative cross-linking within the degrading matrix, driving cell wall stiffening that physically restricts cell expansion and stunts root and shoot elongation. Under a low-vigour state, the shifting coordination of soluble free phenolics no longer reflects metabolic partitioning, but rather signals a non-functional oxidative response associated with widespread membrane degradation and enzymatic dysfunction, directly underpinning the final developmental limitations manifested during early seedling establishment [19].

Conclusively, internal coordination within the antioxidant matrix reflects a critical indicator of the transition from physiological quiescence to terminal metabolic arrest and biophysical destabilisation. In a high-vigour state, this network is characterised by functional partitioning, marked by an inverse association between *α*- and *δ*-tocopherol isomers alongside positive coordination between *β*+*γ*-tocopherols and *β*-carotene. Physiologically, this inverse relationship implies that *δ*-tocopherol potentially operates as a biosynthetic substrate for the *α*-isomer to sustain membrane integrity [37], ensuring that lipophilic agents remain segregated to maintain a ready-response state for germination [39].

Conversely, advanced ageing drives a systemic erosion of these regulated relationships and the emergence of forced molecular proximity. The breakdown of the *α*- and *δ*-tocopherol-tocopherol inverse correlation signals unravelling protection, leaving vulnerable membranes unprotected [37,64]. More critically, the aged matrix features profound co-segregation within the lipid phase, demonstrated by near-absolute alignment between *α*-tocopherol and the lutein + zeaxanthin pool alongside selective degradation of *β*+*γ*-tocopherols. This transition reflects patterns indicative of macromolecular confinement within the relaxing glassy matrix, potentially restricting lateral diffusion and preventing scavengers from intercepting peroxyl radicals [6,37]. Ultimately, the redirection of TAC and *β*-carotene coordination likely mirrors degradation product accumulation under an amorphous solid state [57], thereby intimating an incapacitated antioxidant system unable to neutralise ROS upon imbibition, culminating in the collapse of redox homeostasis and limited radicle protrusion [65].

The functional integrity of the lipophilic antioxidant matrix aligns closely with the performance of the developing seedling, as observed in our previous study [19]. In a high-vigour state, the native abundance of *β*-carotene displays an inverse association with initial seedling elongation, yet strongly supports biomass accumulation, indicating a functional trade-off where these antioxidants prioritise lipid bilayer preservation over rapid cellular extension. Conversely, during prolonged ageing, this regulated protective framework is permanently lost. The accelerated oxidative cleavage and degradation of the *β*-carotene shield directly account for the loss of its positive coordination with biomass accumulation, tracking the extensive membrane damage and enzymatic dysfunction that impair subsequent shoot development [19].

#### 3.3.2. Inter-Network Integration: Multidimensional Mapping of Systemic Physiological Collapse

The metabolic cross-talk and interdependence among the proteomic, carbohydrate, and antioxidant networks directly govern the transition from a high-vigour to a low-vigour state during decadal cold storage. This cascading degradation indicates that terminal viability loss is driven by a system-wide, irreversible collapse of cellular compartmentalisation, permanently compromising the structural and metabolic apparatus required for subsequent germinative activation [65]. The resolution of distinct metabolic trajectories serves to illustrate how specific constitutive baseline profiles drive different pathways of natural ageing. To resolve these complex interactions, the integrated multivariate analysis isolates the systemic decoupling pattern of protein–sugar–phenolic–antioxidant networks under 37 years of natural ageing, providing network-level mechanistic insights into glassy matrix reprogramming and the structural collapse of cellular exclusion boundaries.

Within the spectrum of seed deterioration, high germination energy and germination capacity are sustained through two distinct, constitutive biochemical modalities of defence. To fully capture the distinct physiological strategies inherent to these diverse maize landraces, the first configuration represents a state of active antioxidant readiness, where superior germination parameters track alongside a primed environment characterised primarily by carotenoid pools, while TAC and FPs serve as secondary coordinated indicators within this vital domain—as prominently manifested by fresh seed lots 2, 3 and 4 [62]. This alignment suggests that immediate metabolic readiness and the velocity of radicle protrusion rely on a functional lipophilic shield to prevent lipid peroxidation during the early phases of imbibition. Conversely, the second high-vigour modality is anchored by constitutive proteomic stability, where the total protein pool closely tracks the dominant metabolic agility of the highly soluble albumin fraction, representing a well-preserved structural matrix where essential enzymes and repair proteins remain unconstrained by structural decay, while the globulin and glutelin fractions align as baseline structural components available to fuel embryonic growth upon subsequent rehydration—anchoring the specific baseline of fresh seed lot 1 [7,23]. The complexity of the high-vigour state further reveals that these underlying biochemical matrices manifest as distinct, constitutive physiological determinants that dictate the seed-to-seedling transition. The prime metabolic trajectory is characterised by maximum physiological vigour, supported by a highly coordinated alliance of cell wall structural stabilisers such as IBPs and bound FA [59], being closely paralleled by an embryo-centric biochemical constitution that prioritises a targeted narrow defence involving a tight alignment between globulins and *α*-tocopherol to ensure localised protection of the embryo and lipid-rich membrane components—uniquely resolved as a conserved genetic strategy in fresh seed lot 3 [37,62]. Finally, operating independently of these defensive modalities, a third metabolic trajectory functions at a high-turnover metabolic regime characterised by an accelerated metabolic flux and intensive hexose turnover; while this maximises initial germination energy through rapid carbohydrate mobilisation, it occurs at the expense of long-term biophysical preservation, ultimately predisposing the intracellular matrix to advanced macromolecular decay and isolating the highly distinctive metabolic signature of fresh seed lot 1 along the glucose and fructose vectors [27]. Collectively, these divergent baseline profiles underscore the intrinsic value of these germplasm accessions in exhibiting distinct, genetically underpinned physiological trajectories rather than a uniform response.

In response to prolonged cold storage, the transition to a low-vigour, aged state triggers a pronounced collapse of this organised physiology, revealing highly divergent physiological trajectories among the evaluated germplasm. The earliest stage of physiological decline is characterised by the exhaustion of external sentinels and the onset of structural uncoupling within the dry cytoplasm. This transitional phase is marked by a pronounced compartmentalisation disruption, where the progressive molecular relaxation of the matrix over decadal storage drives the autolytic cleavage of ester bonds anchoring hydroxycinnamic acids to cell wall polysaccharides, leading to the significant accumulation of free *p*-CoumA and FPs—characterising the transitional phase of aged seed lots 3 and 4 [59,60]. Crucially, the spatial mobilisation of secondary *β*+*γ*- and *δ*-tocopherol isomers within this destabilised intracellular environment underscores their role as a critical lipophilic defensive barrier that preserves membrane lipid structures only after the primary cell wall and pigment shields are compromised—uniquely acting as the final lipophilic shield in aged seed lots 3 and 4 [64]. As deterioration progresses, the intracellular environment undergoes a profound catabolic transition—a critical tipping point where the cell shifts from quiescent protection to active catabolic dysregulation and advanced structural decay. This phase is characterised by the extensive proteolytic degradation of storage reserves [48], led by the irreversible physical locking and structural collapse of the glutelin fraction into water-insoluble aggregates, which acts as a primary metabolic driver of structural matrix immobilisation—retaining a highly specific, isolated pattern of structural retention in aged seed lot 1 [35]. Concurrently, intense oxidative stress drives a sharp pattern inversion where broad-spectrum stabilisers are replaced by the progressive proliferation of highly reactive hexose debris and free ferulic acid. This unregulated liberation of reducing sugars generates a severe physiological hazard, accelerating non-enzymatic glycosylation and Maillard-type cross-linking with neighbouring proteins—driving the terminal cellular disintegration of aged seed lot 2, and the spontaneous hexose release in aged seed lot 4 [54,55]. The powerful physiological opposition between these degradation markers and the functional germination traits illustrates the severe energetic and developmental cost of this systemic biopolymer matrix collapse. This systemic failure highlights the unique mechanistic value of evaluating 37-year-term natural ageing within a gene bank, distinct from the acute thermal and hygroscopic shock imposed during accelerated ageing (AA) or controlled deterioration tests (CDT). Under artificial CDT/AA regimes, high temperatures disrupt cellular integrity through rapid enzymatic denaturation and moisture-driven lipid autolysis, often masking the structural dynamics of the glassy state. In contrast, natural decadal ageing operates within a vitrified cytoplasm, where slow biophysical matrix relaxation allows for the gradual, non-enzymatic cleavage of cell wall components and the progressive accumulation of reactive hexose debris over decades. This fundamental difference explains prominent discrepancies in phenolic and carbohydrate dynamics compared to artificial protocols—where rapid transcriptional cascades, hormonal signalling deregulation, and active enzymatic degradation pathways dominate seed ageing kinetics [66]—thereby validating that long-term naturally stored germplasm offers an irreplaceable physiological template for genuine, time-dependent seed ageing.

Ultimately, the advanced aged state culminates in terminal physiological exhaustion, characterised by the absolute collapse of metabolic maintenance and the extensive disintegration of the endosperm’s structural organisation. Within this state of terminal deterioration, the complete uncoupling of functional germination traits from structural integrity is consistent with the hypothesis that the mechanical failure of cellular barriers allows water molecules and reactive substrates to regain local mobility, permanently compromising tissue defence [25,36]. The divergent trajectories among the aged landraces demonstrate that while the biophysical breakdown of the intracellular matrix is an inevitable consequence of time, the specific pathway to exhaustion is ultimately modulated by the unique biochemical profile of each individual maize landrace [52,67], consistently distinguishing the accessions that robustly maintain their characteristic profiles over time. While aged seed lot 1 bypasses comprehensive lateral collapse by retaining its position near the storage reserve vectors, aged seed lot 2 undergoes a state of terminal contraction driven by massive lipid degradation and free ferulic acid accumulation, whereas aged seed lots 3 and 4 shift toward stress-induced phenolic and tocopherol mobilisation. As these exclusion boundaries fail and the structural lipophilic fractions become disrupted, including the complete collapse of TAC coordination which remains fundamentally uncoupled from baseline physiological vitality, the total loss of metabolic control permanently halts the kinetic activation required for successful germinative activation, leaving the embryonic axis entirely vulnerable to lethal oxidative deregulation and lipid peroxidation upon subsequent imbibition [32,33,45].

#### 3.3.3. Study Limitations

While this study presents an integrated framework of biochemical reorganisations during decadal seed storage, all proposed physiological pathways, matrix reorganisations, and cellular decay dynamics are based on correlation networks and multivariate integration. Direct biophysical or subcellular validation experiments—such as glass transition temperature (*T_g_*) measurements, microscopy of subcellular structures, or quantification of specific protein oxidative modifications—were not performed; hence, mechanisms involving macromolecular confinement, oxidative deregulation, and glassy matrix reprogramming remain testable hypotheses.

Moreover, although the accessions studied displayed distinct metabolic trajectories over 37 years of natural ageing, analysing a targeted set of genotypes cannot fully represent the entire genetic variability of a large germplasm collection. Consequently, whilst key biochemical fractions emerged as potential indicators for estimating the extent of functional disintegration of seed defences and subsequent intracellular destabilisation, their formal evaluation as diagnostic markers requires predictive modelling across broader populations. Crucially, a potential limitation regarding year-to-year environmental confounding was effectively eliminated, as the postzygotic phase was strictly maintained within optimal physiological thresholds across both regeneration seasons. Therefore, the observed biochemical shifts directly reflect progressive, storage-induced ageing throughout prolonged cold storage, even though the specific degradation kinetics remain bound to the evaluated genotypes and storage regimes.

## 4. Materials and Methods

### 4.1. Plant Materials and Growth Conditions

The Maize Research Institute Zemun Polje (MRIZP) gene bank active maize collection is maintained under medium-term storage conditions at +4 °C, an approximate relative humidity of 40%, and a seed moisture content of 11%, in strict compliance with international guidelines for the effective management of germplasm collections [11].

This study evaluates four introduced maize landraces (Table 2). Each accession provided two distinct seed lots for comparative physiological and biochemical analyses: the original seed lot harvested in 1985, representing 37 years of ex situ cold storage (aged seed samples; CS1), and its respective counterpart freshly regenerated and harvested in 2022, which served as the baseline physiological comparator (control seed samples; CS0). The detailed passport data and regeneration histories for all evaluated germplasms are summarized in Table 2.

**Figure 6:**
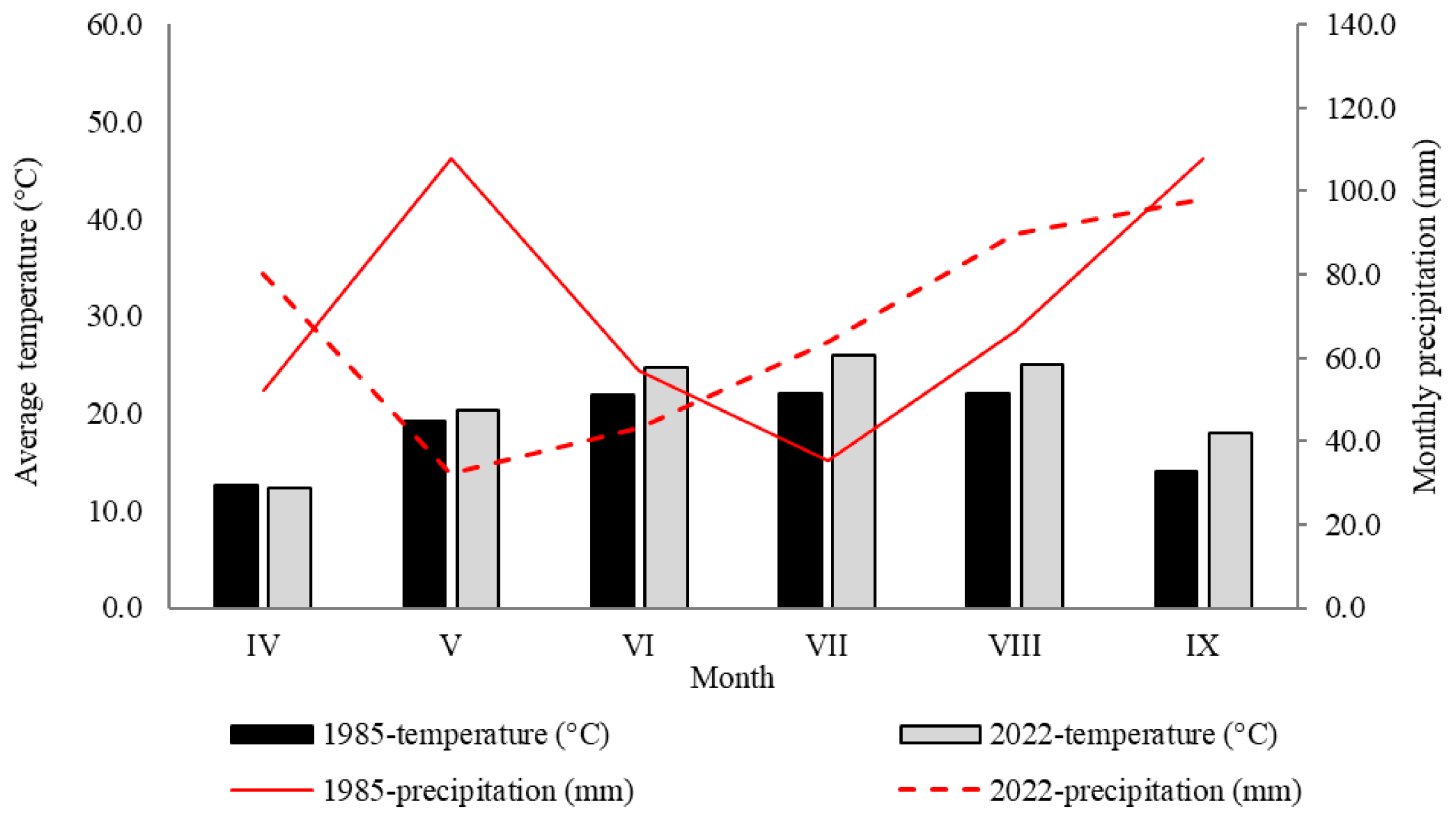
Monthly climatic variables at Zemun Polje during the 1985 and 2022 maize regeneration seasons.

The genotypes (eight seed samples in total) were regenerated at Zemun Polje, Serbia (20°18′60.00″ E, 44°51′59.99″ N, 80 m a.s.l.), characterized by a soil type of Calcaric Chernozem with a silty loam texture. To minimize genetic drift and maintain the integrity of the landraces, the manual multiplication was conducted via controlled pair crossing (full-sibling mating). Technical isolation was strictly applied to prevent gene flow. Standard cropping practices, including weed, pest, and disease control, were applied in both 1985 and 2022. The agro-ecological conditions during the vegetative period (April–September) were optimal for maize growth in both years (Figure 6), meeting the regional precipitation requirement of approximately 459.0 L m^−2^ [68]. The introduced late-maturing genotypes underwent critical subsequent phenophases—the flowering (anthesis and silking in late July) and the grain filling (in August)—under optimal and highly comparable agro-climatic conditions in both 1985 and 2022. Specifically, the primary period of seed component accumulation (August) was characterised by optimal conditions: moderate average temperatures (≈22 °C in 1985 vs. 25 °C in 2022) and abundant precipitation in both production seasons (≈65 mm in 1985 and ≈90 mm in 2022). Environmental variables during both regeneration seasons remained safely within the optimal physiological thresholds for maize growth, without thermal or water deficit stress that could induce confounding maternal environmental effects. Ears were manually harvested at the complete physiological maturity (R6 phenophase).

### 4.2. Assays for Seed Germination Potential

Standard germination tests were conducted in accordance with the International Seed Testing Association (ISTA) rules [69] to determine germination energy (GE) and germination capacity (GC). The assays were performed on 50 seeds per accession with three biological replicates. Seeds were surface-sterilised in 1% sodium hypochlorite for 8 min and rinsed thrice with sterile distilled water. The between-paper method was employed; the moistened papers were rolled and placed into sealed polyethylene bags to maintain constant humidity. Incubation was conducted at 20/30 °C under an 8/16 h light/dark photoperiod for 7 days.

Germination evaluation followed strict ISTA morphological criteria, based on the synchronised growth of essential embryonic structures. Seedlings with abnormal morphology (e.g., deformed coleoptiles, spiral mesocotyls, or stunted primary roots) were classified as abnormal and excluded. Normal seedlings were identified by the emergence of the first foliage leaf from the coleoptile and a well-developed root system (comprising the primary root and lateral secondary roots). Following ISTA protocols, GE (an indicator of seed vigour reflecting the rate and uniformity of seedling emergence) was assessed after 4 days (first count), while GC (representing the potential of seeds to develop into normal seedlings) was evaluated after 7 days (final count). The percentages of GE and GC were calculated following the methodology outlined by Zhang et al. [70]:

(1) $$\mathrm{GE}\;(\%)=(\frac{n_{1}}{N})\times 100\%,$$

(2) $$\mathrm{GC}\;(\%)=(\frac{n_{2}}{N})\times 100\%,$$

where *n*_1_ is the number of normally germinated seeds on Day 4, *n*_2_ is the number of normal seedlings on Day 7, and *N* is the total number of seeds per replicate.

### 4.3. Biochemical Data Sourcing and Integration

While the absolute baseline biochemical profiles utilised in this framework were sourced from Kravic et al. [19], the present study introduces a novel integrative approach. By quantitatively linking these 23 biochemical markers with standardised seed germination metrics (Day 4 first count and Day 7 final count), this work transitions from baseline characterisation to uncovering the dynamic metabolic networks and functional cross-talk governing seed vigour during multi-decadal storage. To elucidate the intra-network interactions and functional coordination underlying seed longevity and physiological deterioration, comprehensive biochemical parameter profiles were consolidated from previously validated datasets [19] analysed herein. This integrated repository encompasses total protein content [71] and storage protein fractions [72], quantified via the Micro-Kjeldahl method [71] and modified Osborne solubility profiling as described by Lookhart and Bean [72], respectively. Additionally, mono- and disaccharides were determined using High-Performance Liquid Chromatography (HPLC) coupled with Refractive Index Detection (HPLC–RID) [73,74].

The metabolic framework further includes soluble-free and insoluble-bound phenolic compounds and individual phenolic acids [75], which were characterised through the Folin–Ciocalteu reagent [76] and HPLC–PDA/RID [77], respectively. Furthermore, the lipophilic antioxidant fraction incorporates carotenoid profiles, comprising *β*-carotene and lut+zeax [78], which were analysed via HPLC coupled with Photodiode Array Detection (HPLC–PDA) [79]. This fraction also includes tocopherol isomers [80], which were quantified by HPLC coupled with Fluorescence Detection (HPLC–FLD). Finally, the total antioxidant capacity was assessed using the ABTS reagent via the direct QUENCHER method [81]. Analytical assays (chromatographic and spectrophotometric measurements) were conducted in technical triplicates and averaged to obtain a single value per biological replicate. The detailed suite of analytical protocols—including primary methodological references [71,72,73,74,75,76,77,78,79,80,81], specific solvent systems, extraction efficiencies, chromatographic columns, mobile phases, and detection parameters for each of the 23 biochemical markers—is detailed extensively by Kravic et al. [19].

### 4.4. Statistical Analysis

The consolidated biochemical datasets [19] were statistically analysed using a two-way factorial analysis of variance (ANOVA) according to a randomized complete block design (RCBD). Significant differences between accession means were determined by Fisher’s least significant difference (LSD) test, with differences at the *p* ≤ 0.05 probability level considered significant. The R software, R version 4.6.0., was used for all statistical analysis [82]. For transparency and context, the mean values and standard deviations of the biochemical parameters are provided in Supplementary Tables S1–S4.

To visualize the distribution, density, and variability of the germination data, violin plots were generated, with kernel density estimation representing the data distribution and individual replicates displayed as internal points. Furthermore, to evaluate the inter-relations between seed germination potential (GE, GC) and the biochemical markers, correlation matrices were computed, and Pearson correlation coefficients (r) were calculated using the R software, package version 2.6.4. [83]. A Principal Component Analysis (PCA) was performed on scaled data [83] to assess the intra-relations of the biochemical framework and to identify the primary biochemical drivers of physiological divergence between the high-vigour CS0 and low-vigour CS1 seed lots. Individual PCAs biplots for germination potential vs. proteomic, carbohydrate, phenolic and antioxidant pools, respectively, are provided in Supplementary Figures S1–S4, along with the factor loadings (Supplementary Tables S5–S8). Prior to conducting the PCA, all original data were standardised using the z-score transformation method in order to eliminate the effects of different measurement units among the physiological and biochemical markers. To avoid pseudoreplication, correlation matrices and PCA models were executed on the dataset comprising *n* = 12 independent biological observations (4 landraces × 3 biological replicates) per physiological state. Statistical significance of Pearson’s r was evaluated at *df* = *n* − 2 = 10 degrees of freedom. The contributions of variables (%) to the principal components (PCs) within the integrated PCA biplots are provided in Supplementary Table S9. All statistical analyses, multivariate modelling, and data visualizations (heatmaps and PCA plots) are performed using the R software environment and its associated statistical packages [82,83].

## 5. Conclusions

The transition from a state of structural integrity to one of a systemic physiological collapse of cellular compartmentation highlights that seed viability is not merely a quantitative measure of stored reserves, but a dynamic manifestation of molecular spatial partitioning. Rather than a stochastic, uniform decline, our integrated multivariate analysis demonstrates that 37 years of natural ageing triggers a systemic decoupling of protein–sugar–phenolic–antioxidant networks. The resulting sample grouping effectively delineates the boundaries separating passive matrix stabilisation from active, embryo-centric protective networks. Crucially, these coordinated macromolecular shifts provide indirect evidence of glassy matrix alterations. The uncoupling of germination potential from the carbohydrate axis points to a critical threshold of cellular stability, where the breakdown of cellular boundaries signifies a terminal loss of metabolic coordination, marking the definitive point of matrix stabilisation failure. By delivering these network-level insights and bridging the gap between static metrics and dynamic failure, this study provides an integrative, physiologically grounded framework to enhance the long-term monitoring and conservation management of stored maize germplasm.

## Figures and Tables

**Table 1:** Principal component analysis (PCA) factor loadings for germination potential and the integrated biochemical profile evaluated across high-vigour (CS0) and low-vigour (CS1) maize seed lots.

Parameters	PC1	PC2	PC3	PC4
Eigenvalue	7.87 ^1^	6.74	4.60	2.98
Variance (%)	31.49	26.96	18.44	11.95
Cumulative variance (%)	31.49	58.46	76.90	88.85
Traits (Factor Loadings)				
Germination energy (GE)	**0.856**	0.401	0.181	−0.085
Germination capacity (GC)	**0.790**	0.570	0.054	0.052
Total protein	**0.809**	−0.389	0.215	0.079
albumins	**0.927**	−0.098	0.167	0.129
globulins	0.504	−0.254	−0.663	−0.420
glutelins	0.441	**−0.886**	−0.042	−0.074
*α*-zeins	0.161	0.510	**0.797**	0.087
glucose	0.120	0.620	0.423	−0.526
fructose	−0.442	−0.012	0.586	−0.643
sucrose	0.138	−0.470	−0.215	0.642
maltose	0.673	−0.047	0.164	0.387
total soluble-free phenolics (FPs)	0.518	0.458	0.055	0.565
free *p*-Coumaric acid (f *p*-CoumA)	**−0.785**	0.160	0.338	0.016
free ferulic acid (f FA)	**−0.735**	−0.270	0.550	0.077
free caffeic acid (f CA)	0.271	**0.771**	−0.260	0.450
total insoluble-bound phenolics (IBPs)	0.108	−0.603	**0.730**	0.243
bound *p*-Coumaric acid (b *p*-CoumA)	**0.715**	−0.635	0.031	0.180
bound ferulic acid (b FA)	−0.135	−0.555	**0.732**	0.326
bound caffeic acid (b CA)	0.498	−0.215	0.433	−0.680
α-tocopherol	**0.776**	−0.183	−0.184	−0.359
*β*+*γ*-tocopherols	−0.210	**0.915**	0.185	0.108
*δ*-tocopherol	0.109	**0.736**	0.442	−0.132
*β*-carotene	0.444	**0.772**	−0.288	0.237
lut + zeax	**0.770**	0.567	−0.164	0.016
Total Antioxidant Capacity (TAC)	0.327	0.144	**0.841**	0.332

Factor loadings > |0.700| are shown in bold. ^1^ Principal components retained according to Kaiser’s criterion (Eigenvalue > 1); Minus signs (−) denote negative correlations.

**Table 2:** Passport data and regeneration history for the evaluated Maize Research Institute Zemun Polje (MRIZP) gene bank maize accessions. Legend: CO–Country of Origin; YR–Year of Regeneration; CSD–Cold Storage Duration (years); GD–Group Designation; CS1–aged seed sample; CS0–control, fresh seed sample; MEX–Mexico; ARG–Argentina.

Sample	ID No.	Accession Name	CO	YR	CSD	GD
1	MRIZP13509	Illinois composite	MEX	1985	37	CS1
2	MRIZP13509	Illinois composite	MEX	2022	0	CS0
3	MRIZP13695	BK × ETO-2007 × 00	ARG	1985	37	CS1
4	MRIZP13695	BK × ETO-2007 × 00	ARG	2022	0	CS0
5	MRIZP13687	BK × ETO-2004 × 08	ARG	1985	37	CS1
6	MRIZP13687	BK × ETO-2004 × 08	ARG	2022	0	CS0
7	MRIZP13706	BK × ETO-1995 × 07	ARG	1985	37	CS1
8	MRIZP13706	BK × ETO-1995 × 07	ARG	2022	0	CS0

## Data Availability

The data presented in this study are available in the article.

## Supplementary Materials

The following supporting information can be downloaded at: https://www.mdpi.com/article/10.3390/plants15162437/s1.

## Abbreviations

The following abbreviations are used in this manuscript:

MRIZP	Maize Research Institute Zemun Polje
CS	cold storage
GE	germination energy
GC	germination capacity
FPs	soluble-free phenolics
IBPs	insoluble-bound phenolics
*p*-CoumA	*p*-Coumaric acid
FA	ferulic acid
CA	caffeic acid
TAC	Total Antioxidant Capacity
ROS	Reactive Oxygen Species
ISTA	International Seed Testing Association
HPLC	High-Performance Liquid Chromatography
PDA	Photodiode Array Detection
RID	Refractive Index Detection
FLD	Fluorescence Detection
