# Supplementary material for: Intra-Relations of Biochemical Profiles Defining the Germination Potential of Medium-Term Cold-Stored Maize (Zea mays L.) Seeds
Source: Plants (Basel). 2026 Aug 11;15(16):2437. doi: 10.3390/plants15162437 (PMC13516885; doi:10.3390/plants15162437)
Supplement: Supplementary file 1 [file plants-15-02437-s001.zip › Figure S2.pdf]

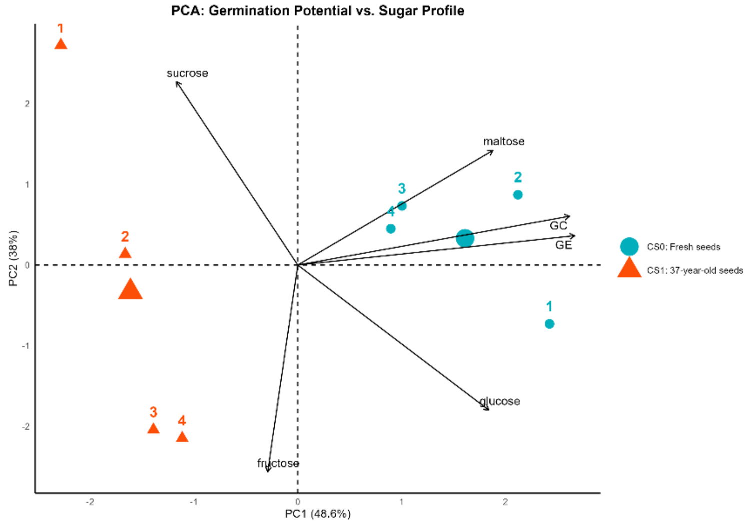


**Figure S2.** PCA biplot illustrating the coordination between germination potential and the carbohydrate matrix in fresh (CS0; blue circles) and 37-year-old (CS1; red triangles) maize seed lots. Vectors indicate factor loadings for the integration of physiological and biochemical variables; numbers (1–4) identify individual maize landrace accessions. Abbreviations: GE–Germination energy; GC–Germination capacity.
