# Supplementary material for: Intra-Relations of Biochemical Profiles Defining the Germination Potential of Medium-Term Cold-Stored Maize (Zea mays L.) Seeds
Source: Plants (Basel). 2026 Aug 11;15(16):2437. doi: 10.3390/plants15162437 (PMC13516885; doi:10.3390/plants15162437)
Supplement: Supplementary file 1 [file plants-15-02437-s001.zip › Table S1.pdf]

Table S1. Total protein and proteins fractions content (% dry matter) in evaluated maize seed lot samples.

Sample	total protein		albumins		globulins		glutelins		α-zeins	
1	12.90±0.20	a	0.765±0.02	c	1.225±0.02	b	2.915±0.02	a	2.915±0.02	f
2	12.93±0.00	a	1.090±0.03	a	1.255±0.01	a	2.755±0.06	b	3.370±0.03	b
3	11.79±0.01	f	0.515±0.02	e	1.125±0.01	c	2.420±0.03	c	3.060±0.06	e
4	11.93±0.01	e	0.740±0.01	c	1.150±0.01	c	2.335±0.02	d	3.300±0.01	c
5	11.70±0.04	f	0.425±0.04	f	1.280±0.01	a	2.310±0.01	d	2.860±0.03	g
6	12.37±0.11	c	0.650±0.03	d	1.265±0.01	a	2.440±0.03	c	3.100±0.01	d
7	12.04±0.04	d	0.365±0.04	g	1.015±0.05	d	2.295±0.06	d	3.650±0.01	a
8	12.65±0.04	b	0.855±0.01	b	1.140±0.01	c	2.205±0.08	e	3.670±0.01	a
CV (%)	0.48		2.68		1.28		1.32		0.60	
LSD	0.1026		0.0314		0.0261		0.0562		0.0337	

Legend: CV—coefficient of variation; LSD—least significant difference. The results are mean ± SD of independent biological replicates (n = 3, each analysed in technical triplicates) integrated from Kravic et al. (2025) [19]. Means followed by the same letter within the same column are not significantly different ($p \leq 0.05$). Samples 1, 3, 5, and 7—the original 37-year old samples, CS1 (from 1985); samples 2, 4, 6, and 8—the control fresh samples, CS0 (newly regenerated in 2022). The seed samples belong to the following maize genotypes: MRIZP13509 (samples 1, and 2); MRIZP13695 (samples 3, and 4); MRIZP13687 (samples 5, and 6); MRIZP13706 (samples 7, and 8).
