# Supplementary material for: Intra-Relations of Biochemical Profiles Defining the Germination Potential of Medium-Term Cold-Stored Maize (Zea mays L.) Seeds
Source: Plants (Basel). 2026 Aug 11;15(16):2437. doi: 10.3390/plants15162437 (PMC13516885; doi:10.3390/plants15162437)
Supplement: Supplementary file 1 [file plants-15-02437-s001.zip › Table S2.pdf]

Table S2. Reducing and non-reducing sugars content (% dry matter) in evaluated maize seed lot samples.

Sample	glucose		fructose		sucrose		maltose	
1	0.467±0.00	d	0.188±0.00	g	1.520±0.04	a	1.089±0.01	c
2	0.692±0.00	a	0.366±0.01	c	1.072±0.01	c	1.277±0.02	b
3	0.522±0.00	c	0.269±0.00	d	1.173±0.01	b	0.842±0.00	de
4	0.697±0.01	a	0.230±0.00	f	1.203±0.02	b	1.441±0.34	a
5	0.656±0.00	b	0.395±0.00	b	1.088±0.18	c	0.671±0.00	f
6	0.658±0.05	b	0.184±0.01	g	1.216±0.01	b	0.956±0.05	cd
7	0.713±0.00	a	0.439±0.00	a	1.172±0.00	b	0.762±0.01	ef
8	0.641±0.02	b	0.253±0.01	e	1.247±0.02	b	0.915±0.03	d
CV (%)	2.18		1.27		3.89		8.80	
LSD	0.0238		0.0064		0.0811		0.1514	

Legend: CV—coefficient of variation; LSD—least significant difference. The results are mean ± SD of independent biological replicates (n = 3, each analysed in technical triplicates) integrated from Kravic et al. (2025) [19]. Means followed by the same letter within the same column are not significantly different ($p \leq 0.05$). Samples 1, 3, 5, and 7—the original 37-year old samples, CS1 (from 1985); samples 2, 4, 6, and 8—the control fresh samples, CS0 (newly regenerated in 2022). The seed samples belong to the following maize genotypes: MRIZP13509 (samples 1, and 2); MRIZP13695 (samples 3, and 4); MRIZP13687 (samples 5, and 6); MRIZP13706 (samples 7, and 8).
