# Supplementary material for: Intra-Relations of Biochemical Profiles Defining the Germination Potential of Medium-Term Cold-Stored Maize (Zea mays L.) Seeds
Source: Plants (Basel). 2026 Aug 11;15(16):2437. doi: 10.3390/plants15162437 (PMC13516885; doi:10.3390/plants15162437)
Supplement: Supplementary file 1 [file plants-15-02437-s001.zip › Table S3.pdf]

Table S3. (**a**) Total soluble-free phenolic compounds (FPs) content (μg GAE g^{-1} d.m.) and soluble-free phenolic acids content (μg g^{-1} d.m.) in evaluated maize seed lot samples. (**b**) Total insoluble-bound phenolic compounds (IBPs) content (μg GAE g^{-1} d.m.) and insoluble-bound phenolic acids content (μg g^{-1} d.m.) in evaluated maize seed lot samples.

**a**								
**Sample**	**total FPs**		**free *p*-CoumA**		**free FA**		**free CA**	
1	219.71±15.1	c	3.73±0.07	c	1.69±0.22	c	0.01±0.00	c
2	211.37±22.5	c	1.56±0.30	e	1.04±0.09	de	0.01±0.00	c
3	319.28±34.7	a	4.49±0.00	b	2.18±0.04	b	2.56±0.13	b
4	315.55±18.0	a	3.75±0.13	c	1.25±0.22	d	3.51±0.49	b
5	248.60±12.1	b	4.63±0.09	b	1.30±0.17	cd	0.01±0.00	c
6	218.68±19.4	b	4.69±0.09	b	0.64±0.04	e	3.83±0.26	ab
7	259.75±13.6	b	5.05±0.17	a	3.54±0.04	a	0.01±0.00	c
8	267.45±32.8	c	3.18±0.21	d	0.76±0.13	e	5.34±0.26	a
CV (%)	6.18		2.91		15.18		5.39	
LSD	27.5282		0.1957		0.4071		1.7771	
**b**								
**Sample**	**total IBPs**		**bound *p*-CoumA**		**bound FA**		**bound CA**	
1	2454.3±138.2	ab	331.26±3.64	a	2412.32±11.81	b	1.88±0.08	c
2	2486.3±85.6	a	287.06±6.47	b	2370.91±1.09	c	2.56±0.19	a
3	2340.9±71.1	bc	188.61±6.90	e	2307.14±10.84	d	1.54±0.02	d
4	2329.1±185.5	bc	224.39±5.41	d	2264.17±2.80	e	1.62±0.15	d
5	2157.9±56.3	d	192.59±10.44	e	2027.14±12.84	g	1.89±0.08	c
6	2169.3±40.7	d	242.73±10.74	c	1992.43±4.01	h	2.11±0.00	b
7	2454.4±26.2	ab	193.29±8.76	e	2478.78±11.78	a	2.17±0.04	b
8	2245.6±121.7	cd	230.90±2.89	d	2120.10±22.07	f	1.89±0.13	c
CV (%)	3.15		2.22		0.36		3.88	
LSD	127.0554		9.0923		14.1746		0.1315	

Legend: *p*-CoumA — *p*-coumaric acid; FA — ferulic acid; CA — caffeic acid; GAE—gallic acid equivalent; CV—coefficient of variation; LSD—least significant difference. The results are mean ± SD of independent biological replicates ($n = 3$, each analysed in technical triplicates) integrated from Kravic et al. (2025) [19]. Means followed by the same letter within the same column are not significantly different ($p \leq 0.05$). Samples 1, 3, 5, and 7—the original 37-year old samples, CS1 (from 1985); samples 2, 4, 6, and 8—the control fresh samples, CS0 (newly regenerated in 2022). The seed samples belong to the following maize genotypes: MRIZP13509 (samples 1, and 2); MRIZP13695 (samples 3, and 4); MRIZP13687 (samples 5, and 6); MRIZP13706 (samples 7, and 8).
