# Supplementary material for: Intra-Relations of Biochemical Profiles Defining the Germination Potential of Medium-Term Cold-Stored Maize (Zea mays L.) Seeds
Source: Plants (Basel). 2026 Aug 11;15(16):2437. doi: 10.3390/plants15162437 (PMC13516885; doi:10.3390/plants15162437)
Supplement: Supplementary file 1 [file plants-15-02437-s001.zip › Table S4.pdf]

Table S4. Tocopherols and carotenoids content (μg g^{-1} d.m.), and Total Antioxidant Capacity (TAC) (μmol Trolox g^{-1} d.m.) in evaluated maize seed lot samples.

Sample	*α*-T		*β*+*γ*-T		*δ*-T		*β*-carotene		lut+zeaxanth		TAC	
1	8.23±0.46	c	10.18±0.57	f	0.59±0.03	f	0.35±0.00	f	9.06±0.12	e	20.11±0.18	bcd
2	10.76±0.42	b	22.38±0.87	e	1.40±0.05	e	2.61±0.07	d	21.52±0.43	d	21.03±0.04	ab
3	4.24±0.21	f	39.93±1.94	c	0.43±0.12	g	1.79±0.05	e	4.97±0.10	h	19.72±1.42	cde
4	4.29±0.21	f	41.63±2.17	c	2.02±0.10	c	16.87±0.49	a	22.61±0.43	c	20.491±0.03	abc
5	5.25±0.23	e	32.11±1.46	d	1.59±0.08	d	2.91±0.08	d	6.43±0.13	f	18.68±0.11	e
6	12.12±0.54	a	39.40±1.75	c	1.44±0.06	e	15.58±0.45	b	35.44±0.54	a	19.06±0.88	de
7	5.09±0.26	e	45.68±2.33	b	2.32±0.12	b	0.33±0.04	f	5.83±0.13	g	21.02±0.00	ab
8	7.12±0.37	d	58.90±3.12	a	2.86±0.15	a	14.33±0.42	c	32.01±0.51	b	21.52±0.48	a
CV (%)	3.56		3.73		4.31		4.11		1.43		3.07	
LSD	0.4393		2.3409		0.1178		0.4874		0.4271		1.0731	

Legend: T— tocopherol; CV—coefficient of variation; LSD—least significant difference. The results are mean ± SD of three independent biological replicates (n = 3, each analysed in technical triplicates) integrated from Kravic et al. (2025) [19]. Means followed by the same letter within the same column are not significantly different ($p \leq 0.05$). Samples 1, 3, 5, and 7—the original 37-year old samples, CS1 (from 1985); samples 2, 4, 6, and 8—the control fresh samples, CS0 (newly regenerated in 2022). The seed samples belong to the following maize genotypes: MRIZP13509 (samples 1, and 2); MRIZP13695 (samples 3, and 4); MRIZP13687 (samples 5, and 6); MRIZP13706 (samples 7, and 8).
