# Supplementary material for: Intra-Relations of Biochemical Profiles Defining the Germination Potential of Medium-Term Cold-Stored Maize (Zea mays L.) Seeds
Source: Plants (Basel). 2026 Aug 11;15(16):2437. doi: 10.3390/plants15162437 (PMC13516885; doi:10.3390/plants15162437)
Supplement: Supplementary file 1 [file plants-15-02437-s001.zip › Table S5.pdf]

Table S5. Principal component analysis (PCA) factor loadings for germination potential and the proteomic matrix across high-vigour (CS0) and low-vigour (CS1) maize seed lots.

**Parameters**	**PC1**	**PC2**
Eigenvalue	3.44 [1]	2.20
Variance (%)	49.09	31.39
Cumulative variance (%)	49.09	80.48
Traits (Factor Loadings)		
Germination energy (GE)	**0.896**	–0.327
Germination capacity (GC)	**0.807**	–0.428
total protein	**0.798**	0.229
albumins	**0.966**	0.075
globulins	0.418	**0.701**
glutelins	0.413	**0.776**
α-zeins	0.258	**–0.877**

Factor loadings > |0.700| are shown in bold. [1] Principal components retained according to Kaiser's criterion (Eigenvalue > 1); Minus signs (–) denote negative correlations.
