# Supplementary material for: Intra-Relations of Biochemical Profiles Defining the Germination Potential of Medium-Term Cold-Stored Maize (Zea mays L.) Seeds
Source: Plants (Basel). 2026 Aug 11;15(16):2437. doi: 10.3390/plants15162437 (PMC13516885; doi:10.3390/plants15162437)
Supplement: Supplementary file 1 [file plants-15-02437-s001.zip › Table S6.pdf]

Table S6. Principal component analysis (PCA) factor loadings for germination potential and the carbohydrate matrix across high-vigour (CS0) and low-vigour (CS1) maize seed lots.

Parameters	PC1	PC2
Eigenvalue	2.92 [1]	2.28
Variance (%)	48.62	38.04
Cumulative variance (%)	48.62	86.66
Traits (Factor Loadings)		
Germination energy (GE)	**0.964**	0.311
Germination capacity (GC)	**0.946**	0.218
glucose	0.665	–0.649
fructose	–0.104	**–0.926**
sucrose	–0.422	**0.821**
maltose	0.679	0.513

Factor loadings > |0.700| are shown in bold. [1] Principal components retained according to Kaiser's criterion (Eigenvalue > 1); Minus signs (–) denote negative correlations.
