# Supplementary material for: Intra-Relations of Biochemical Profiles Defining the Germination Potential of Medium-Term Cold-Stored Maize (Zea mays L.) Seeds
Source: Plants (Basel). 2026 Aug 11;15(16):2437. doi: 10.3390/plants15162437 (PMC13516885; doi:10.3390/plants15162437)
Supplement: Supplementary file 1 [file plants-15-02437-s001.zip › Table S7.pdf]

Table S7. Principal component analysis (PCA) factor loadings for germination potential and the phenolic matrix across high-vigour (CS0) and low-vigour (CS1) maize seed lots.

**Parameters**	**PC1**	**PC2**
Eigenvalue	3.84 [1]	3.41
Variance (%)	38.42	34.08
Cumulative variance (%)	38.42	72.51
Traits (Factor Loadings)		
Germination energy (GE)	**0.919**	0.097
Germination capacity (GC)	**0.899**	–0.136
total soluble-free phenolics (FPs)	–0.371	–0.584
free *p*-Coumaric acid (f *p*-CoumA)	–0.685	–0.535
free ferulic acid (f FA)	**–0.876**	0.274
free caffeic acid (f CA)	0.474	**–0.704**
total insoluble-bound phenolics (IBPs)	–0.227	**0.856**
bound *p*-Coumaric acid (b *p*-CoumA)	0.417	0.664
bound ferulic acid (b FA)	–0.451	**0.732**
bound caffeic acid (b CA)	0.397	0.687

Factor loadings > |0.700| are shown in bold. [1] Principal components retained according to Kaiser's criterion (Eigenvalue > 1); Minus signs (–) denote negative correlations.
