# Supplementary material for: Intra-Relations of Biochemical Profiles Defining the Germination Potential of Medium-Term Cold-Stored Maize (Zea mays L.) Seeds
Source: Plants (Basel). 2026 Aug 11;15(16):2437. doi: 10.3390/plants15162437 (PMC13516885; doi:10.3390/plants15162437)
Supplement: Supplementary file 1 [file plants-15-02437-s001.zip › Table S8.pdf]

Table S8. Principal component analysis (PCA) factor loadings for germination potential and the antioxidant matrix across high-vigour (CS0) and low-vigour (CS1) maize seed lots.

Parameters	PC1	PC2
Eigenvalue	4.34 [1]	1.89
Variance (%)	54.22	23.65
Cumulative variance (%)	54.22	77.87
Traits (Factor Loadings)		
Germination energy (GE)	**0.884**	–0.283
Germination capacity (GC)	**0.957**	–0.185
α-tocopherol	0.421	**–0.796**
β+γ-tocopherols	0.520	**0.731**
δ-tocopherol	0.644	0.622
β-carotene	**0.843**	0.056
lut+zeax	**0.940**	–0.250
Total Antioxidant Capacity (TAC)	0.426	0.396

Factor loadings > ǀ0.700ǀ are shown in bold. [1] Principal components retained according to Kaiser's criterion (Eigenvalue > 1); Minus signs (–) denote negative correlations.
