# Supplementary material for: Intra-Relations of Biochemical Profiles Defining the Germination Potential of Medium-Term Cold-Stored Maize (Zea mays L.) Seeds
Source: Plants (Basel). 2026 Aug 11;15(16):2437. doi: 10.3390/plants15162437 (PMC13516885; doi:10.3390/plants15162437)
Supplement: Supplementary file 1 [file plants-15-02437-s001.zip › Table S9.pdf]

Table S9. Contribution of variables (%) to the first two principal components (PC1 and PC2). Legend: PC1 and PC2 represent the first and second principal components, respectively. Values indicate the percentage of variance contributed by each parameter to the respective component. Total Contribution represents the weighted average contribution across both dimensions.

a

Parameters	PC1	PC2	Total contribution
Germination Energy (GE)	23.36	4.89	14.12
Germination Capacity (GC)	18.94	8.33	13.64
total protein	18.55	2.39	10.47
albumins	27.15	0.26	13.70
globulins	5.09	22.38	13.74
glutelins	4.97	26.70	15.84
α-zeins	1.94	35.05	18.49

b

Parameters	PC1	PC2	Total contribution
Germination Energy (GE)	31.85	0.75	16.30
Germination Capacity (GC)	30.68	2.09	16.39
glucose	15.16	18.48	16.82
fructose	0.38	37.58	18.98
sucrose	6.11	29.55	17.83
maltose	15.82	11.54	13.68

c

Parameters	PC1	PC2	Total contribution
Germination Energy (GE)	21.98	0.28	11.13
Germination Capacity (GC)	21.06	0.55	10.80
total soluble-free phenolics (FPs)	3.59	10.00	6.80
free *p*-Coumaric acid (f *p*-CoumA)	12.22	8.38	10.30
free ferulic acid (f FA)	20.02	2.20	11.11
free caffeic acid (f CA)	5.87	14.58	10.22
total insoluble-bound phenolics (IBPs)	1.35	21.48	11.41
bound *p*-Coumaric acid (b *p*-CoumA)	4.52	12.95	8.73
bound ferulic acid (b FA)	5.30	15.73	10.51
bound caffeic acid (b CA)	4.11	13.84	8.97

d

Parameters	PC1	PC2	Total contribution
Germination Energy (GE)	18.02	4.23	11.12
Germination Capacity (GC)	21.11	1.81	11.46
α-tocopherol	4.09	33.49	18.79
β+γ-tocopherols	6.23	28.26	17.24
δ-tocopherol	9.57	20.46	15.02
β-carotene	16.38	0.17	8.28
lut+zeax	20.38	3.30	11.84
Total Antioxidant Capacity (TAC)	4.21	8.28	6.25

e

Parameters	PC1	PC2	Total contribution
Germination Energy (GE)	9.32	2.38	5.85
Germination Capacity (GC)	7.92	4.83	6.38

total protein	8.32	2.25	5.28
albumins	10.92	0.14	5.53
globulins	3.22	0.96	2.09
glutelins	2.47	11.64	7.06
α-zeins	0.33	3.85	2.09
glucose	0.18	5.70	2.94
fructose	2.49	0.00	1.25
sucrose	0.24	3.27	1.76
maltose	5.75	0.03	2.89
total soluble-free phenolics (FPs)	3.41	3.11	3.26
free *p*-Coumaric acid (f *p*-CoumA)	7.83	0.38	4.11
free ferulic acid (f FA)	6.86	1.08	3.97
free caffeic acid (f CA)	0.93	8.83	4.88
total insoluble-bound phenolics (IBPs)	0.15	5.40	2.77
bound *p*-Coumaric acid (b *p*-CoumA)	6.49	5.98	6.24
bound ferulic acid (b FA)	0.23	4.57	2.40
bound caffeic acid (b CA)	3.15	0.69	1.92
α-tocopherol	7.65	0.50	4.07
β+γ-tocopherols	0.56	12.42	6.49
δ-tocopherol	0.15	8.04	4.10
β-carotene	2.51	8.86	5.68
lut+zeax	7.54	4.76	6.15
TAC	1.36	0.31	0.84
